# The pentose phosphate pathway constitutes a major metabolic hub in pathogenic *Francisella*

**DOI:** 10.1371/journal.ppat.1009326

**Published:** 2021-08-02

**Authors:** Héloise Rytter, Anne Jamet, Jason Ziveri, Elodie Ramond, Mathieu Coureuil, Pauline Lagouge-Roussey, Daniel Euphrasie, Fabiola Tros, Nicolas Goudin, Cerina Chhuon, Ivan Nemazanyy, Fabricio Edgar de Moraes, Carlos Labate, Ida Chiara Guerrera, Alain Charbit

**Affiliations:** 1 Université de Paris, Paris, France; 2 INSERM U1151 - CNRS UMR 8253, Institut Necker-Enfants Malades. Team 7: Pathogénie des Infections Systémiques, Paris, France; 3 Pole Bio-analyse d’images, Structure Fédérative de Recherche Necker INSERM US24- CNRS UMS 3633, Paris, France; 4 Plateforme Protéome Institut Necker, PPN, Structure Fédérative de Recherche Necker INSERM US24-CNRS UMS 3633, Paris, France; 5 Plateforme Etude du métabolisme, Structure Fédérative de Recherche Necker INSERM US24-CNRS UMS 3633, Paris, France; 6 Laboratório Max Feffer de Genética de Plantas, Departamento de Genética, Escola Superior de Agricultura Luiz de Queiroz, Universidade de São Paulo, Piracicaba, Brazil; Emory University School of Medicine, UNITED STATES

## Abstract

Metabolic pathways are now considered as intrinsic virulence attributes of pathogenic bacteria and thus represent potential targets for antibacterial strategies. Here we focused on the role of the pentose phosphate pathway (PPP) and its connections with other metabolic pathways in the pathophysiology of *Francisella novicida*. The involvement of the PPP in the intracellular life cycle of *Francisella* was first demonstrated by studying PPP inactivating mutants. Indeed, we observed that inactivation of the *tktA*, *rpiA* or *rpe* genes severely impaired intramacrophage multiplication during the first 24 hours. However, time-lapse video microscopy demonstrated that *rpiA* and *rpe* mutants were able to resume late intracellular multiplication. To better understand the links between PPP and other metabolic networks in the bacterium, we also performed an extensive proteo-metabolomic analysis of these mutants. We show that the PPP constitutes a major bacterial metabolic hub with multiple connections to glycolysis, the tricarboxylic acid cycle and other pathways, such as fatty acid degradation and sulfur metabolism. Altogether our study highlights how PPP plays a key role in the pathogenesis and growth of *Francisella* in its intracellular niche.

## Author summary

Metabolic pathways are intimately associated with the virulence of pathogenic bacteria and are therefore interesting antibacterial targets. Here we focused on the role of the pentose phosphate pathway (PPP) in the intracellular life cycle of the bacterium *Francisella novicida*. We were able to show, by combining genetic and imaging approaches, that this pathway was a major contributor to the intracellular survival of the bacterium. Complementary proteomics and metabolomics approaches revealed that PPP was at the crossroads of many other metabolic pathways. This work has thus allowed us to better understand the key role played by the PPP in the survival of *Francisella* in its intracellular niche.

## Introduction

*Francisella tularensis* is the causative agent of the zoonotic disease tularemia [1]. This facultative intracellular bacterial pathogen is able to infect numerous cell types but is thought to replicate and disseminate mainly in macrophages *in vivo* [2]. The four major subspecies (subsp) of *F*. *tularensis* currently listed are the subsps: *tularensis*, *holarctica*, *mediasiatica* and *novicida* (the latter is also called *F*. *novicida*). These subsps differ in their virulence and geographical origin [3] but all cause a fulminant disease in mice that is similar to tularemia in humans [4]. Although *F*. *novicida* is rarely pathogenic in humans, its genome shares a high degree of nucleotide sequence conservation with the human pathogenic subsp *tularensis* and is thus widely used as a model to study highly virulent subspecies.

*Francisella* virulence is tightly linked to its capacity to multiply exclusively in the cytosolic compartment of infected cells, and in particular in macrophages in vivo. Cytosolic pathogens, that notably include *Listeria monocytogenes* and *Shigella flexneri*, often require the utilization of multiple host-derived nutrients [5–7] and hexoses are generally their preferred carbon and energy sources. The capacity of *Francisella* to multiply in the host cytosol is controlled by multiple regulatory circuits [8], connected to metabolism. In particular, we have shown that gluconeogenesis was essential for *Francisella* intracellular multiplication [9,10], allowing host-derived substrates such as amino acid, pyruvate and glycerol to be used as carbon, nitrogen and energy sources.

The pentose phosphate pathway (PPP) constitutes, with glycolysis, a major pathway for glucose catabolism. However, its contribution to bacterial metabolic adaptation and especially its importance in bacterial pathogenesis, remains largely unexplored. The PPP is composed of two branches, an oxidative and a non-oxidative branch [11]. Glucose flux through the oxidative branch produces NADPH, an essential reductant in anabolic processes. The non-oxidative branch generates the five-carbon sugar Ribose-5P (R-5P) from glucose and can be reversibly converted into glycolytic intermediates such as glyceraldehyde 3P (GA-3P) and Fructose-6P (F-6P). *Francisella*, which lacks the oxidative branch of the PPP, is equipped with a complete non-oxidative branch composed of the four enzymes: *tktA* (*FTN_1333*, encoding transketolase), *rpiA* (*FTN_1185*, encoding ribose 5-phosphate isomerase), *rpe* (*FTN_1221*, encoding ribulose phosphate 3-epimerase) and *talA* (*FTN_0781*, encoding transaldolase) (S1 Fig). These four genes are scattered along the *F*. *novicida* chromosome and each of them is present in a single copy.

Transketolase, which is central to the non-oxidative branch of the PPP, can produce either R-5P or Sedoheptulose-7-phosphate (S-7P) in response to available metabolite concentrations. Ribulose-5-phosphate (Ru-5P), can be converted either into xylulose-5- phosphate (Xyl-5-P) by Rpe or R-5P by RpiA, respectively. Finally, TalA catalyzes the conversion of S-7P and GA-3P to erythrose-4-phosphate (E-4P) and fructose-6-phosphate (F-6P). Overall, the different precursors synthesized by the non-oxidative branch of the PPP contribute to multiple important functions of the bacterial cell, including biosynthesis of lipopolysaccharide (LPS), aromatic amino acids and nucleic acid precursors.

Here, we performed a functional analysis of the four PPP mutants (*tktA*, *rpe*, *talA* and *rpiA*) and a global approach based on the analysis of their proteo-metabolomic features to highlight PPP-related pathways and potential biological hubs. The data presented suggest a biphasic bacterial regulation mode between glycolysis and PPP during intracellular multiplication, and reveal previously unrecognized links between PPP and other metabolic pathways.

## Results

### Transketolase, a conserved enzyme of the PPP

Transketolase enzymes are ubiquitously expressed in eukaryotes and bacteria. In bacteria, they allow the production of precursors required for the synthesis of nucleotides and certain amino acids as well as for LPS synthesis (S1 Fig). *Francisella* genomes possess a unique transketolase-encoding gene (here designated *tktA*). The transketolase TktA of *F*. *novicida* (FTN_1333) is a 663 amino acid long protein that shows 41% to 57% amino acid sequence identity with its orthologs in other pathogenic bacterial species (*eg*. it shares 55.6% and 41% amino acid identity with the transketolases of *Legionella pneumophila* and *Mycobacterium tuberculosis*, respectively). It should be noted that several bacteria express multiple isoforms of transketolases. For example, *Escherichia coli* has two genes (*tktA* and *tktB*), *Salmonella typhimurium* has three genes (*tktA*, *tktB*, and *tktC*), encoding transketolases with different enzymatic properties, and *Citrobacter rodentium* genomes encode up to six transketolase isoforms.

**Fig 1:**
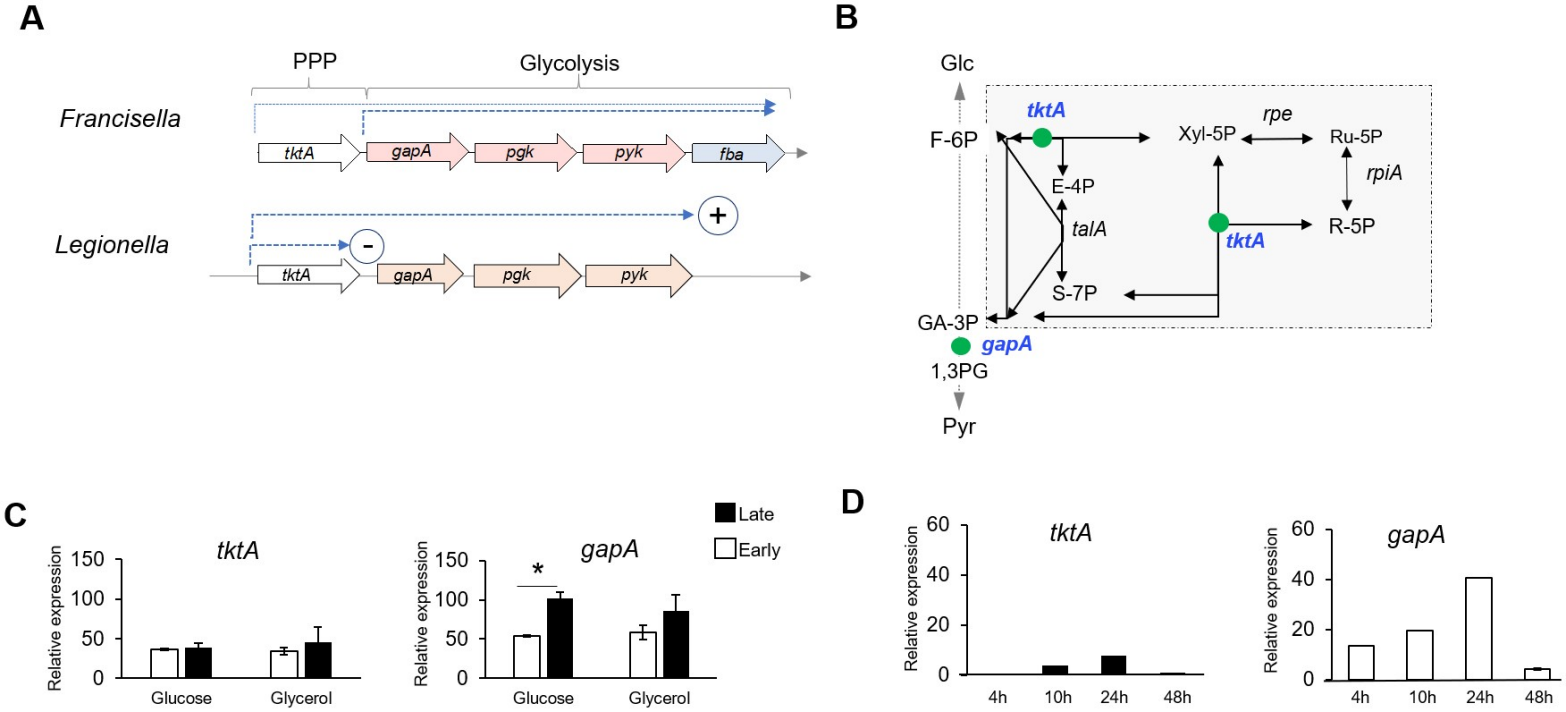
Transcriptional analysis of *tktA* and *gapA* genes. **(A)** Schematic organization of the *tktA* operon in *F*. *novicida* and *Legionella pneumophila*. The terms PPP and Glycolysis on top, indicate the genes involved in either the PPP or the Glycolytic/gluconeogenic pathways. The last gene of the *Francisella* locus is absent in the *Legionella* locus (*fba*). The dotted blue arrows indicate the predicted transcriptional units. In *Legionella*, in the absence of the CsrA regulatory protein, transcription is interrupted after *tktA* (circled sign -) whereas in the presence of CsrA, transcription resumes till the end of the operon (circled sign +). (**B**) The enzymatic reactions corresponding to TktA and GapA enzymes are shown as green balls on a schematic depiction of the PPP and glycolytic pathways. **(C)** qRT-PCR analysis of *tktA* and *gapA* genes in WT *F*. *novicida*, grown in CDM supplemented either with glucose or glycerol, in exponential (Early, white labels) and stationary phase of growth (Late, black labels). **(D)** qRT-PCR analysis of *tktA* and *gapA* genes in WT *F*. *novicida*, over a 24 hour-period of intracellular growth in J774-1 macrophages. *, P <0.01 (as determined by Student’s t test).

In *F*. *tularensis* species, *tktA* is the first gene of a highly conserved operon [10] and precedes genes (*gapA*, *pgK*, *pyK* and *fba*, respectively) involved in glycolysis/gluconeogenesis (Fig 1A and 1B). The organization of the four first genes of this operon (*tktA-pyK*) is conserved in both *L*. *pneumophila* [12] and *Coxiella burnetii* species (S2 Fig and S1 Table). Several other pathogenic bacterial species, such as *Bordetella pertussis* and *Brucella melitensis*, also have *tktA*, *gapA* and *pgk* genes in the same genetic cluster and with the same organization but lack the *pyk* and *fba* genes. The species with most medical relevance were chosen to be depicted in the figure showing the *tktA* operon genetic context (S2 Fig). Notably, in most *Burkholderia* species (including *B*. *multivorans*, *B*. *pseudomallei*, *B*. *thailandensis*, …), the *tktA* and *gapA* genes are adjacent and in the same orientation, suggesting that they belong to the same transcriptional unit, whereas the *pgk*, *pyk*, and *fba* genes cluster is located in a distinct region of the chromosome.

These observations prompted us to first quantify the transcription of the two consecutive *F*. *novicida* genes *tktA* and *gapA* by qRT-PCR, in wild-type (WT) bacteria grown in chemically defined medium (CDM) [13]. Transcription of each gene was found to be approximately similar in the presence of glucose or glycerol, suggesting that their expression is not controlled by these carbon sources. (Fig 1C). The expression of the *gapA* gene was consistently higher than that of the *tktA* gene with both carbon sources. Of note, *gapA* gene expression appeared to be higher in late exponential phase (OD_600nm_ of 1–1.2) than in early exponential phase (OD_600nm_ of 0.5), while *tktA* gene expression remained unchanged.

Transcription of *tktA* and *gapA* genes was next quantified in J774.1-infected macrophages during a 48 h-period (Fig 1D). Expression of both genes progressively increased during the first 24 h of infection (corresponding to the active phase of intracellular bacterial multiplication), and dropped at 48 h. As in CDM, *gapA* gene expression was significantly higher than that of *tktA* at all time-points tested (approximately 5-fold higher), suggesting that *gapA* possesses its own promoter. Although RT-PCR analyses have shown that the genes *tktA* and *gapA* were co-transcribed [10], a promoter prediction analysis of the sequence immediately upstream of the *gapA* gene (BPROM, executed on-line at www.softberry.com) identified putative -35 and -10 promoter elements, sharing significant homology to the consensus site recognized by the general sigma factor σ70 (S1 Fig).

### Importance of the PPP in bacterial growth and intracellular multiplication

Earlier studies have already shown that transketolase mutants of *Francisella* were defective for growth in macrophages and attenuated for virulence in mice [14–17]. To further investigate these results, here we evaluated the impact of inactivation of each of the four PPP genes (*tktA*, *rpe*, *talA* and *rpiA*). The *tktA* mutant was generated by allelic replacement while the *rpiA*, *rpe*, and *talA*, mutants were obtained from the *F*. *novicida*- 2-allele set—mutant library [18] and kindly provided by Anders Sjostedt (S2 Table). The four *F*. *novicida* mutants were designated Δ*tktA*, Δ*rpe*, Δ*rpiA* and Δ*talA* for simplicity.

**Fig 2:**
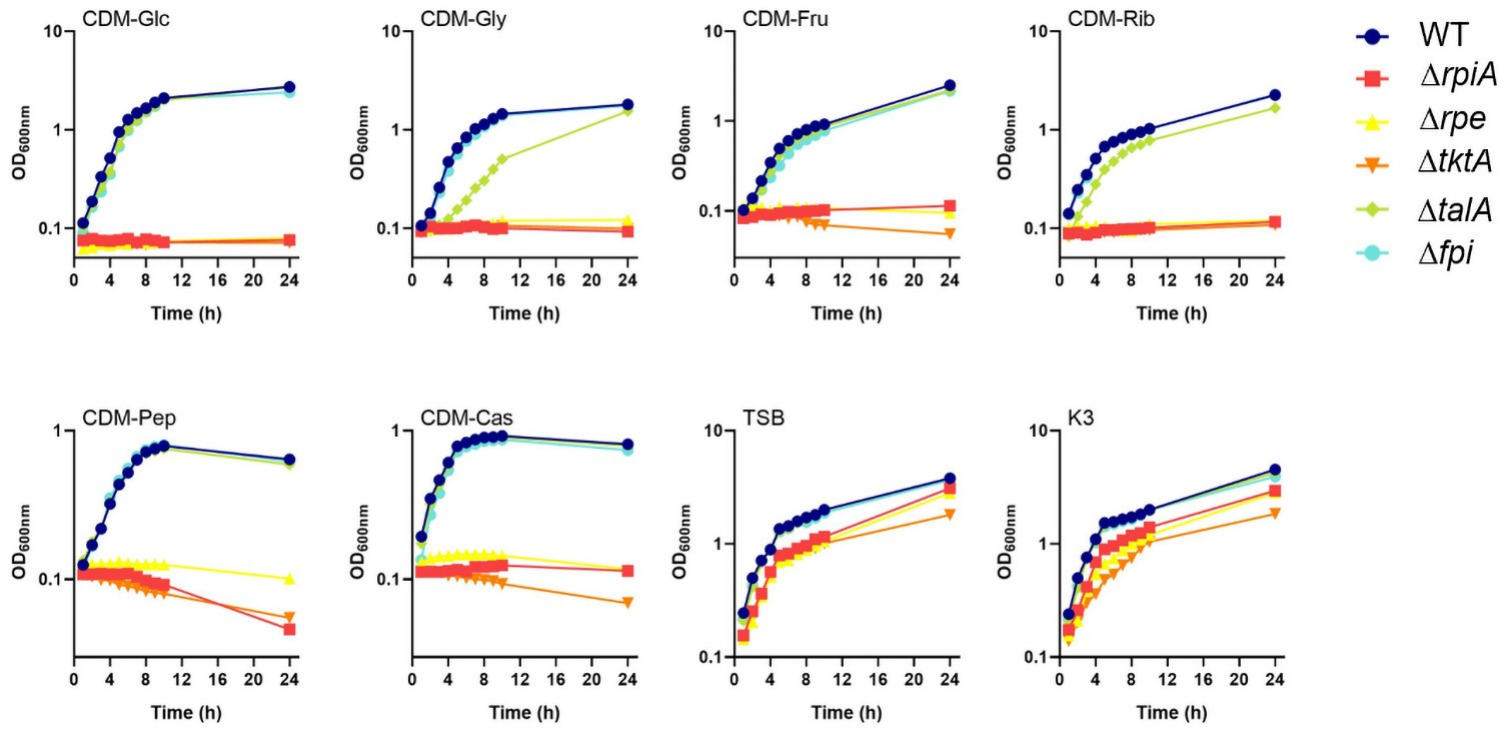
Growth of the PPP mutants in liquid culture. Bacterial growth was monitored in CDM supplemented with various carbon sources: Glc (glucose), Gly (glycerol), Rib (ribose) and Fru (fructose), at a final concentration of 25 mM; or Cas (casamino acids), and Pep (peptone) at a final concentration of 0.2%. Bacterial growth was also monitored in two complex media: TSB (trypic soya broth) and K3 (Shaedler K3 medium). Stationary-phase bacterial cultures of wild-type *F*. *novicida* (WT), Δ*tktA*, Δ*rpE*, Δ*rpiA*, Δ*talA and Δfpi* mutants were diluted to a final OD_600nm_ of 0.1, in 20 mL broth. Every hour, the OD_600nm_ of the culture was measured, during a 24 h-period.

Bacterial growth was first evaluated in CDM supplemented with various carbohydrates, and in two complex media, tryptic soya broth supplemented with cysteine (0.1% w/v) and glucose (0.4% w/v) (TSB) and Schaedler K3 (K3). Three mutants, Δ*tkt*, Δ*rpe* and Δ*rpiA*, failed to grow in any of CDMs tested (Fig 2). Wild-type growth was restored in the three corresponding complemented strains (designated Δ*tktA*-Cp, Δ*rpe*-Cp and Δ*rpiA*-Cp, respectively; see Materials and methods), confirming the lack of polar effect of the mutations (S3A–S3C Fig). The Δ*talA* mutant showed wild-type growth in all the media tested, except in CDM supplemented with glycerol where a very modest growth defect was observed. The fact that in CDM conditions where casamino acids or a complex amino acid mixture (peptone) were provided, the three mutants Δ*tktA*, Δ*rpe* and Δ*rpiA* remained unable to grow, suggest that the nitrogen source in CDM is not dictating the growth defects observed and that the phenotypes are probably solely due to the carbohydrate carbon sources. In complex media (TSB and K3), the four mutants showed wild-type growth, indicating that they do not correspond to essential genes.

**Fig 3:**
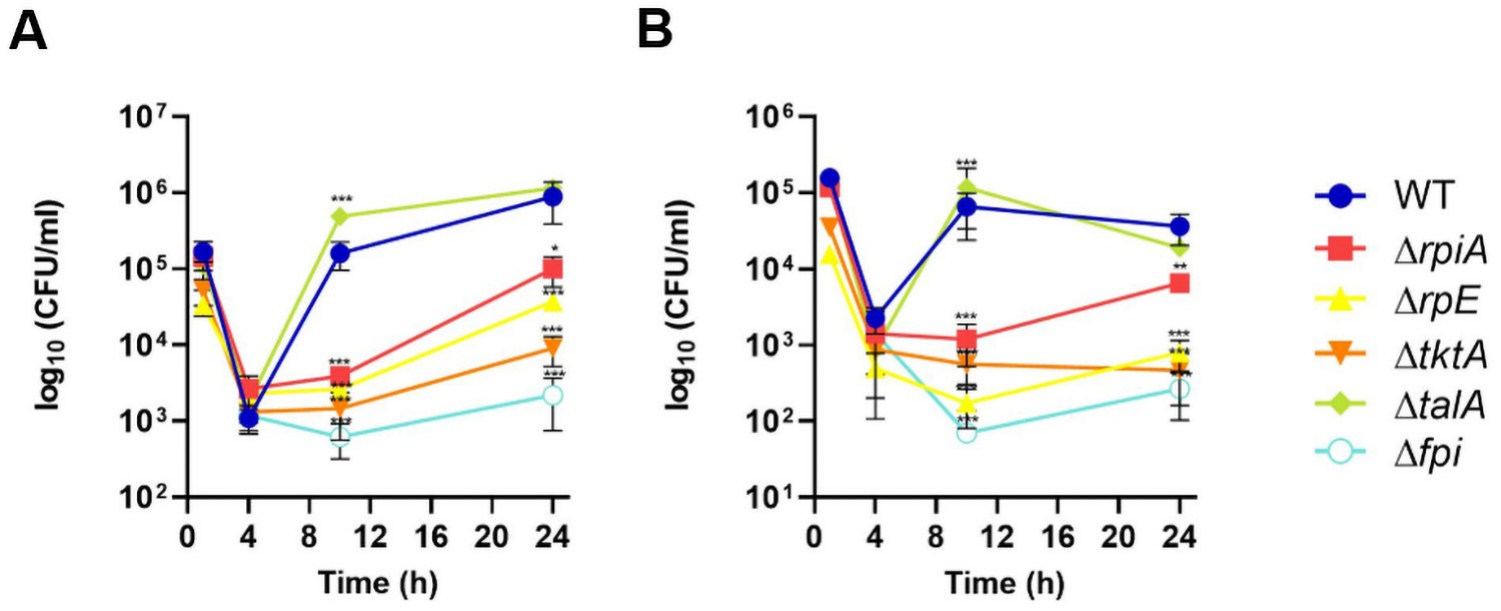
Intracellular multiplication of the PPP mutants. Kinetics of intracellular multiplication of the mutants was monitored in J774.1 macrophages over a 24 h-period in DMEM supplemented with either (**A**) Glucose (Glc), or (**B**) Glycerol (Gly), and compared to that in the wild-type *F*. *novicida* (WT). A Δ*fpi* mutant strain was used as a negative control. *, P <0.05; **, P <0.01; ***, P <0.001 (compared to WT strain; as determined by two-way ANOVA test).

We next monitored intracellular multiplication of the PPP mutants in the murine macrophage-like cell line J774.1. As we previously observed that intracellular multiplication of some *Francisella* mutants could vary depending on the nature of the carbon source present in the cell culture medium [9,10], we monitored growth of the PPP mutants in glucose-supplemented medium (glycolytic substrate) as well as in glycerol-supplemented medium (gluconeogenic substrate) (Fig 3A and 3B).

In agreement with earlier reports, intracellular multiplication of the Δ*tktA* mutant in DMEM-glucose was totally abrogated during the first 10 h after infection and similar to that of the Δ*fpi* mutant lacking the entire *Francisella* pathogenicity island [19]. A modest increase of bacterial counts (colony forming units, CFUs) was however recorded at 24 h, indicating that the Δ*tktA* mutant had started to multiply (an 8-fold increase in CFUs compared to Δ*fpi*). Similar behavior was recorded for the Δ*rpiA* and Δ*rpe* mutants that failed to multiply up to 10 hours post-infection but for which a restart of intracellular multiplication was recorded at 24 h (the CFUs recorded were however still approximately 10- to 100-fold lower than that of WT). However, the CFUs recorded were still approximately 8- to 30-fold lower than that of WT. Functional complementation (Δ*tktA*-Cp, Δ*rpe*-Cp and Δ*rpiA*-Cp strains) always restored wild-type intracellular multiplication (S3D–S3F Fig). In DMEM-glycerol, multiplication of the three mutants was also severely impaired although the Δ*rpiA* mutant showed a slightly better multiplication than the Δ*tktA* and Δ*rpe* mutants. Of note, multiplication of the Δ*talA* mutant was unaffected and even superior to that of the WT strain in both DMEM-glucose and DMEM-glycerol at all time-points tested, suggesting the conservation of a transaldolase activity in this mutant. Since each of the intermediate metabolites of the PPP can theoretically be synthesized in the absence of TalA, this enzyme might be dispensable under most physiological conditions.

To assess a potential defect in bacterial entry into cells, we compared the percentage of entry of the different mutants to that of the wild-type strain after one hour of infection (corresponding to t0 of the intracellular cycle, see Materials and methods). The Δ*tktA*, Δ*ripA*, and Δ*talA* mutants showed similar entry efficiency to the wild-type strain. Only the Δ*rpe* mutant showed a defect in cell entry (30% of the wild-type strain), possibly contributing to the intracellular growth defect observed in this mutant (S4 Fig).

To rule out a possible hyper cytotoxicity of the PPP mutants, we next quantified lactate dehydrogenase (LDH) release in cell culture supernatants. Under the infection conditions we used for CFU counts, only marginal LDH release was recorded at 10 h with the WT strain as well as with the four PPP mutants, demonstrating that none of these mutants were highly cytotoxic. In agreement with earlier reports [20], upon active cytosolic multiplication, *F*. *novicida* induced a cytopathogenic effect at 24 h. The % spontaneous LDH release was 25% in uninfected control cells; it reached 55% in Δ*talA*-infected cells; 45% with WT and Δ*rpiA*-infected cells; 30% with Δ*rpe*-infected cells; and less than 20% with Δ*tktA* and Δ*fpi*-infected cells (S5A Fig). We also measured the % of dead cells by the trypan blue uptake assay (S5B Fig). Only marginal cell death was recorded (<10%) with all the strains tested, further confirming the lack of cytotoxicity of the PPP mutants.

The intracellular behavior of the four PPP mutants was finally tested in bone marrow-derived macrophages (BMMs) from C57BL/6 mice, in DMEM medium supplemented with glucose and 10% of fetal bovine serum (FBS). Comforting the observations in J774-1 cells, growth of the Δ*tktA*, Δ*rpiA* and Δ*rpe* mutants was severely impaired at 10 h (2-log lower counts compared to WT). However, no apparent restart of growth was recorded at 24 h with these mutants, likely due to the higher bactericidal activity of primary macrophages. In contrast, multiplication of the Δ*talA* mutant remained identical to that of WT *F*. *novicida* at all time points tested (S6 Fig).

Overall, these data show that the ability to consume available carbon sources in infected macrophages, which depends on the enzymes TktA, Rpe and RpiA, is essential for intracellular *Francisella* multiplication.

### Dynamics of macrophage infection of the PPP mutants

**Fig 4:**
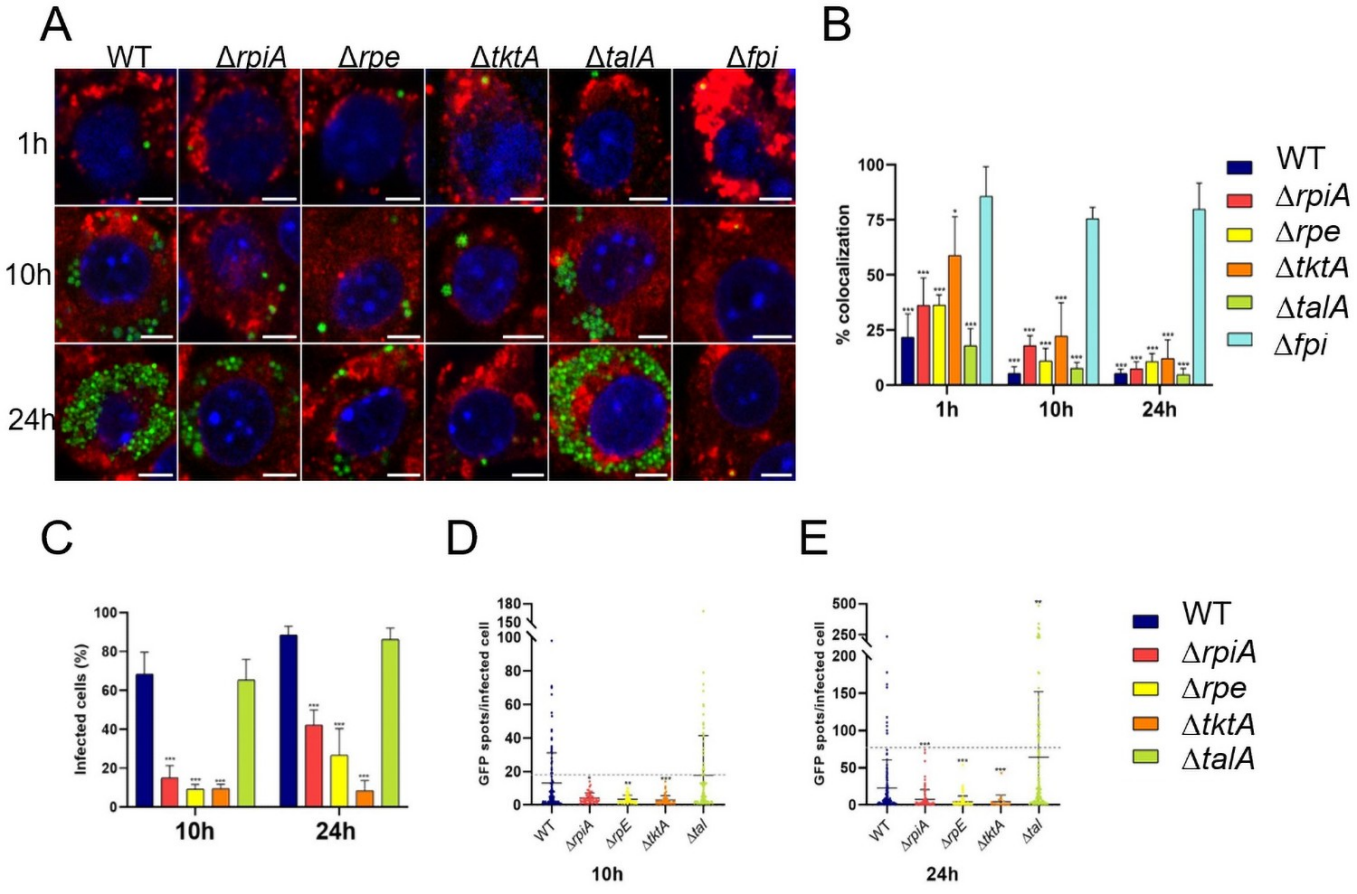
Subcellular localization of the PPP mutants. Glucose-grown J774.1 were infected for 1 h with wild-type *F*. *novicida* (WT), Δ*rpiA*, Δ*rpe*, Δ*tktA*, Δ*talA*, or Δ*FPI* strains and their co-localization with the phagosomal marker LAMP-1 was observed by confocal microscopy 1h, 10h and 24 h, after beginning of the experiment. (**A**) Scale bars at the bottom right of each panel correspond to 5 μm. J774.1 were stained for *F*. *tularensis* (*green*), LAMP-1 (*red*), and host DNA (*blue*, DAPI stained). (**B**) Quantification of bacteria/phagosome colocalization in glucose-grown J774.1 macrophages. Mean and SD of triplicate wells. ***p<0,0001 (compared to Δ*FPI* strain; as determined by ANOVA one-way test). (**C**) Percentage of infected cells at 10 and 24 h was quantified by using imageJ software. At least 1 000 cells per condition were counted. ***p<0,0001 (compared to WT strain; as determined by ANOVA one-way test). The number of GFP-positive spots per infected cell was quantified at 10h (**D**) and 24 h (**E**) by using the Icy Software. We analyzed at least 100 infected cells for each condition. ***p<0,0001 (compared to WT strain; as determined by ANOVA one-way test).

We then used imaging approaches to characterize, at the single cell level, the impact of the four mutations in the PPP. We first monitored by confocal immunofluorescence microscopy the subcellular localization of the four PPP mutants, using GFP-labeled bacteria, and the late phagosomal marker LAMP-1 (Fig 4A and 4B). As expected, the Δ*fpi* negative control strain showed a high colocalization with LAMP-1 at all time points tested (up to 80% at 24 h), confirming that it remained trapped in phagosomes. At 1 h, the % colocalization with LAMP-1 did not exceed 25% with WT and Δ*talA* strains; was approximately 36% for the Δ*rpiA*, and Δ*rpe* mutant strains; and 60% for Δ*tktA*. Thus, at this early stage, for all PPP mutants, the majority of bacteria had already escaped from the phagosomal compartment, with the exception of Δ*tktA* which exhibited a slight escape defect. At 10 h and 24 h, the % colocalization with LAMP-1 remained below 10% with WT and Δ*talA* strains. Confirming their presence in the cytosolic compartment, the Δ*rpiA*, Δ*rpe* and Δ*tktA* mutants showed less than 25% colocalization with LAMP-1 at 10 h (17.9%,10.9% and 22.3% respectively); and less than 15% at 24 h (7.4%, 10.7%, 12.1%, respectively) (Fig 4B).

We also quantified the percentage of infected cells at 10 h and 24 h (Fig 4C). With the WT and Δ*talA* mutant strains, almost 70% of cells were infected at 10 h, while this percentage was below 20% for the three mutants. At 24 h, the percentage of infected cells was above 80% for WT and Δ*talA* mutant strains, and increased to 42% and 27% for Δ*rpiA* and Δ*rpe*, respectively, but remained unchanged with Δ*tktA*. To precise these data, we further quantified the number of GFP-positive spots per infected cell, by counting a total of 100 infected cells per strain, at 10h and 24 h (Fig 4D and 4E). Comparable results were recorded with WT and Δ*talA* strains. At both time-points tested, a broad distribution of the number of GFP-positive spots was recorded. At 10 h, these ranged from 1 to more than 100 spots per infected cell (with a majority of infected cells having less than 20 spots). At 24 h, the number of cells bearing more than 60 spots further increased, in particular with Δ*talA*. In contrast, with the three other PPP mutants, the number of GFP-positive spots recorded was below 15 per infected cell at 10 h and reached 74 and 54 for for Δ*rpiA* and Δ *rpe*, respectively; but remained below 43 for Δ*tktA*, at 24h.

Altogether, these results suggest that the observed intracellular growth defects of PPP Δ*rpiA*, Δ*rpe*, and Δ*tktA* mutants, is primarily a consequence of their inability to cope with the cytosolic compartment environment of infected macrophages.

**Fig 5:**
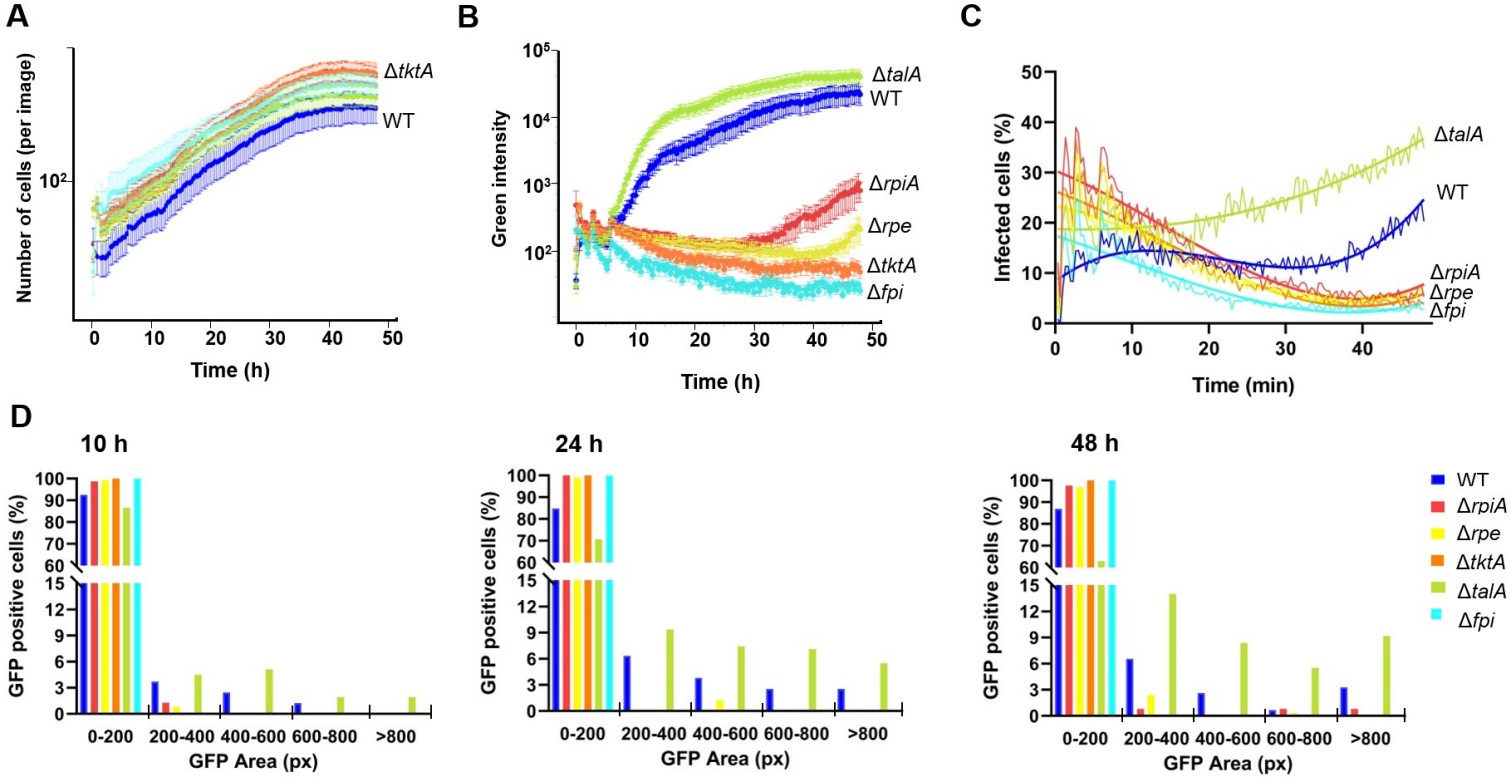
Time lapse video microscopy analyses of the PPP mutants. J774.1_red_ cells (expressing mKate2 nuclear-restricted red fluorescent protein) were infected with bacteria expressing the green fluorescent protein (GFP), in DMEM supplemented with glucose (MOI = 100). One hour after infection, cells were washed several times with gentamicin-containing medium (10 μg mL^-1^) to remove extracellular bacteria. A gentamicin concentration of 10 μg mL^-1^ was then maintained throughout the experiment. Images were acquired every 20 min using the IncuCyte S3 live cell imaging system (Essen BioScience) over a 48-h period. The kinetics of intracellular multiplication of PPP mutants were monitored by Incucyte S3 software (Incucyte Live-Cell Analysis System, Sartorius). *F*. *novicida* WT is shown in blue; the negative control strain Δ*fpi*, in cyan; Δ*rpiA*, in red; Δ*rpe* in yellow; Δ*tktA*, in orange; and Δ*talA*, in green. (**A**) Total number of J774.1_red_ cells was determined by counting the number of red nuclei per image every 20 minutes. An image taken 1h post-infection generally contains between 50 and 100 cells. Results are presented as the mean and standard deviation of sixteen images. (**B**) Bacterial multiplication (GFP-expressing bacteria) was monitored by checking the total area of green particles (*i*.*e*., GFP-expressing bacteria) in each image (containing between 50 and 250 cells) every 20 min. Multiplication was translated into graphical lines using the Incucyte S3 software. Results are presented as the mean and standard deviation of sixteen images. (**C**) The total number of GFP-positive cells (*i*.*e*., infected with GFP-expressing bacteria) was monitored by quantifying the percentage of cells with red nuclei associated with at least one detectable green intensity signal in one image (containing between 50 and 250 cells). Results are presented as the mean and interpolation curve of eight images. (**D**) At selected times (10 h, 24 h, and 48 h), the number of GFP-positive cells was decomposed into 5 categories based on the area of the detected GFP signal (in pixels; px): i) 0–200; px ii) 200–400; px, iii) 400–600; px; iv) 600–800; and >800; px; and corresponding to increasingly infected cells. The data presented correspond to the total number of cells in 8 images.

We next wished to follow and quantify the dynamics of intracellular multiplication of the PPP mutants by time-lapse video microscopy, using a fully automated microscope (Incucyte 531 S3, Essen BioScience). J774–1 macrophages with red nuclei (here designated J774.1_red_) were infected with GFP-expressing bacteria at an MOI of 100 and infection was followed over a 48 h-period, in 96-well plates (Figs 5 and S7 and S1–S5 Movies). We first quantified the total number of J774.1_red_ cells over time to evaluate the impact of bacterial infection on cell viability (see Materials and methods). This assay showed that cells were able to continuously multiply at least during the 40 first hours and were not affected by bacterial infection (Fig 5A). Intracellular bacterial multiplication was next followed by monitoring the total green area intensity (reflecting multiplication of the GFP-positive bacteria) over time translated on graph line by using the Incucyte S3 software (Fig 5B). Multiplication of the Δ*tktA*, Δ*rpe* and Δ*rpiA* mutants was affected to variable extents. The Δ*tktA* mutant was again the most severely affected whereas the Δ*talA* mutant showed wild-type multiplication.

We then used a machine learning approach (see Data analyses, Materials and methods; S8 Fig) to quantify from the video microscopy data: i) the total number of cells, at each time point, and ii) for each cell, at each time point, the presence of a GFP signal (bacteria) and the GFP area (bacterial multiplication).

The total number of J774.1_red_ cells infected with wild-type bacteria increased during the first 10 h of infection, then reached a plateau until 40h and increased again between 40 and 48 h (Fig 5C). This increase was faster and continuous with the Δ*talA* mutant than with the wild-type strain. In contrast with the three PPP mutants, the total number of J774.1_red_ cells infected with GFP-expressing bacteria progressively decreased with time, suggesting a continuous elimination of these mutant bacteria similar to that of the Δ*fpi* negative control strain. At selected time points (10 h, 24 h, and 48 h), the amount of GFP signal detected was accurately quantified (GFP area, see Materials and methods). This analysis revealed that after 10 h, more than 85% of the GFP-positive cells contained few bacteria as indicated by a measured GFP area of less than 200 pixels for all strains tested (estimation range of 1 to 10 bacteria per cell; Fig 5D). However, in 1–2% of cells infected with Δ*rpiA* and Δ*rpe* mutants, bacterial multiplication had occurred, as quantified by measured GFP areas of 200–400 pixels. As expected, wild-type bacteria showed a greater capacity for intracellular multiplication, with measured GFP areas in the 200–400, 400–600 and up to the 600-800-pixel ranges. Remarkably, the Δ*talA* mutant also showed a heterogeneous and even broader bacterial intracellular multiplication capacity than the wild-type strain. After 48 h, 40% of the cells infected with the Δ*talA* mutant showed a GFP area >200 pixels, as compared to only 15% with the wild-type strain. Of note, after 24 h, cells infected with Δ*rpiA* and Δ*rpe* mutants were still identified in the 200–400 pixels range (1–2% of the cells) and for the Δ*rpiA* mutant approximately 2% of the cells were in the >600 pixels range, revealing an active multiplication of this mutant in a limited subset of cells.

Overall, these analyses revealed that Δ*tktA*, Δ*rpe* and Δ*rpiA* mutants had impaired intracellular multiplication until 30 h after infection. The Δ*rpe* and Δ*rpiA* mutants then resumed growth in a limited subset of cells, reaching up to 30% that of WT. A very modest intracellular multiplication of the Δ*tktA* mutant could also be visualized between 40 h and 48 h (Fig 5 and S1–S5 Movies). The Δ*talA* mutant showed wild-type or even improved intracellular multiplication-dissemination, in all the conditions tested.

### Virulence assay in the adult fly

The impact of PPP mutations was then assessed in vivo. For this purpose, we used the adult fly model, a simple animal model that has been used previously to assess the attenuation of virulence of *Francisella* mutants [17,21–24].

We wanted to know if the effect of the mutants on fly survival depended on bacterial proliferation. Therefore, we monitored the survival of flies after infection and bacterial multiplication in infected flies. Adult male flies were infected with wild-type *F*. *novicida* (WT), or Δ*tktA*, Δ*rpiA*, Δ*rpe*, Δ*talA*, and Δ*fpi* isogenic mutants (see Materials and methods).

Fly survival was monitored over a 10-day period, using 20 adult male *Drosophila* per bacterial strain. 100% of the flies infected with the WT strain or the Δ*talA* mutant strain died within 6–7 days while 100% of the flies infected with the Δ*fpi* mutant strain survived. The Δ*tktA*, Δ*rpiA* and Δ*rpe* mutants were only slightly less virulent than the WT and killed 90% of the flies after 10 days (S9A Fig). Hence, in spite of showing impaired intracellular multiplication, the Δ*tktA*, Δ*rpiA* and Δ*rpe* mutants retained most of their virulence properties in this model.

To examine bacterial growth within the adult fly, we used a total of 8 flies per bacterial strain and each assay was performed in triplicate. The average number of bacteria (CFUs) recovered from flies 2 h after pricking varied between 4x10^3^ and 8x10^4^ bacteria/8 flies. By day 4 post-infection, bacterial growth reached 5x10^7^ bacteria/8 flies for the WT strain as well as for the three PPP mutants, whereas the counts of the Δ*fpi* mutant remained below 9x10^6^ bacteria/8 flies. For WT and Δ*talA* strains, CFUs were not monitored after day 4 due to insufficient number of surviving flies (< 24). By day 6 post-infection, bacterial growth reached 1 x 10^8^ bacteria/8 flies for the three PPP mutants, whereas the CFUs of the Δ*fpi* mutant (control) reached only 9x10^6^ bacteria/8 flies. For Δ*rpiA*, Δ*rpE*, Δ*tktA* strains, CFUs were not monitored after day 6 due to insufficient number of surviving flies (<24). By day 8 post-infection, the CFUs of the Δ*fpi* mutant (control) remained approximately 1 x10^7^ bacteria/8 flies. Of note, in this model, the Δ*fpi* mutant was also able to multiply until day 6 and persisted in infected flies at day 8 even if it did not cause any death (S9B Fig).

### Invalidation of PPP key enzymes leads to common variations of the global proteomic profile

Having determined the impact of PPP gene inactivation on *F*. *novicida* intracellular survival and virulence, we then wished to further our understanding of the consequences of the mutations on bacterial physiology and to identify possible links between PPP and other metabolic pathways. To this end, we performed a whole cell proteomic analysis, combined with a global metabolomic analysis of the PPP mutants. Whole cell proteomic approaches provide a global view of the post-transcriptional alterations caused by a mutation. Metabolomic approaches bring complementary information on the metabolic changes generated by these alterations and are particularly relevant when studying mutations in enzymatic pathways.

**Fig 6:**
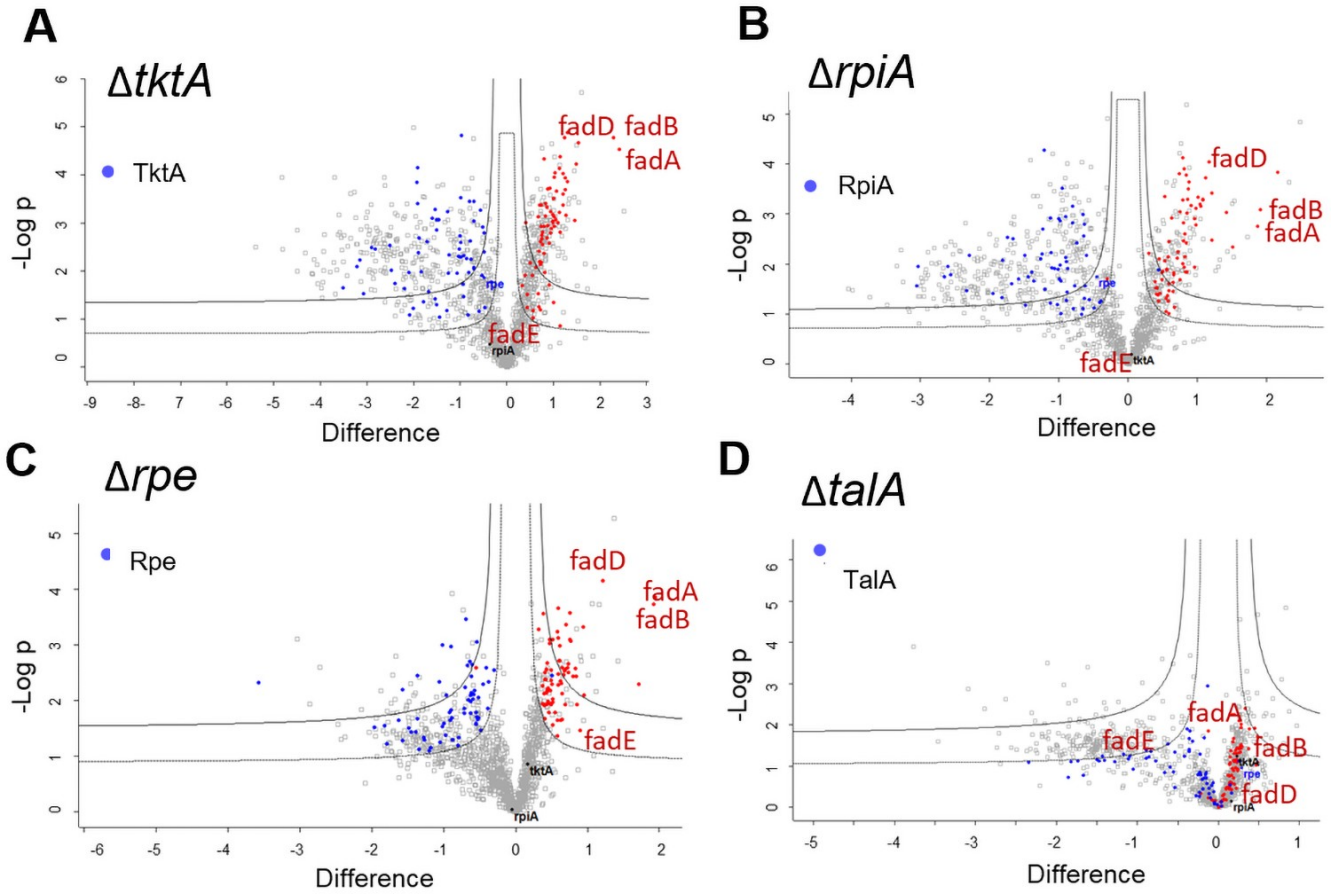
Differential proteomes of WT and Δ*tktA*, Δ*rpe and ΔrpiA* mutants. Bacteria were cultured in TSB and collected during exponential phase of growth (at OD_600_ of 0.5). The proteomes of the four PPP mutants Δ*tktA*, Δ*rpiA*, Δ*rpe* and Δ*talA*, was compared to that of WT *F*. *novicida*. Volcano plot representing the statistical comparison of the protein LFQ intensities of each mutant versus WT. Inner volcano was established using S0 = 0.1, FDR = 0.05 and the outer volcano using S0 = 0.1, FDR = 0.01. The abscissa reports the fold change in logarithmic scale (difference), the ordinate the–log(pvalue). Proteins undergoing the same modulation in Δ*tktA*, Δ*rpe* and Δ*rpiA* mutants but not in Δ*talA* are highlighted in color (blue and red for decreased increased in the mutant, respectively).

We first performed a whole-cell comparative nanoLC-MS/MS proteomic analysis of WT *F*. *novicida* and the three mutant strains that showed impaired intracellular growth (Δ*tktA*, Δ*rpe and ΔrpiA*). As a control we also performed the whole cell comparative analysis of WT *F*. *novicida* and of the Δ*talA* mutant that did not show any intracellular growth defect (Fig 6). Whole cell protein samples were prepared from bacteria grown at 37°C in rich medium (TSB). Each strain was analyzed in three independent biological replicates (see Materials and methods).

In all cases, invalidation of the gene, and therefore absence of the protein, induced a strong modulation of the global amount of protein compared to the WT. Overall, 1 502 proteins were identified across samples. Of those, we retained 1 381 proteins confidently quantified in at least one condition, covering 80% of the proteome on 1 722 proteins predicted to be encoded by the *F*. *novicida* U112 genome. We performed a t-test (FDR<0.05) showing that a large number of proteins was impacted by the deletions: 835 proteins by Δ*tktA*, 690 proteins by Δ*rpiA*, 372 by Δ*rpe* and 237 by Δ*talA* (S3 Table). As expected, for each mutant, the proteins deleted from the genome were found as absent in the corresponding mutant. Of note, in all three mutants Δ*tktA*, Δ*rpiA* and Δ*rpe*, expression of the TalA protein was found increased.

A common set of 145 proteins was modulated in the four mutants. However, an additional set of 137 proteins was modulated in Δ*tktA*, Δ*rpiA*, Δ*rpe* mutants but not in the Δ*talA* mutant which might be involved in their impaired intracellular behavior (Fig 6 and S3 Table). The vast majority of these proteins was following the same modulation pattern, with 73 upregulated and 64 downregulated in the three mutant strains. Enrichment pathway analysis, performed using Kegg mapper (https://www.genome.jp/kegg/mapper.html), highlighted perturbation of proteins mainly involved in metabolic pathways, including the PPP (with 17 proteins downregulated and 34 upregulated) and biosynthesis of secondary metabolites and cofactors (with 23 proteins downregulated and 20 upregulated), suggesting a complex modulation of these pathways. Of note, the amplitude of protein modulation, in terms of fold changes, was stronger in Δ*rpiA* and Δ*tktA*, than in Δ*rpe*.

Interestingly, most of the proteins encoded by the fatty acid degradation locus *fad* (S10 Fig), were up- regulated in the three mutants but not in the Δ*talA* control strain (Fig 6). These results were confirmed by qRT-PCR, indicating that the impact of *tktA* gene inactivation on Fad proteins expression occurred already at the transcriptional level. Indeed, a ca. 3-fold increased expression of most of the genes of the *fad* operon was recorded in exponentially-grown bacteria in K3 medium (OD_600_ 0.5), in the mutant compared to WT. In late exponential phase (OD_600_ 1), this increase reached up to 6-fold that of WT (S10 Fig). Altogether these data strongly suggested a yet unanticipated link between PPP and fatty acid metabolism.

### Metabolomic analyses of the PPP mutants

**Fig 7:**
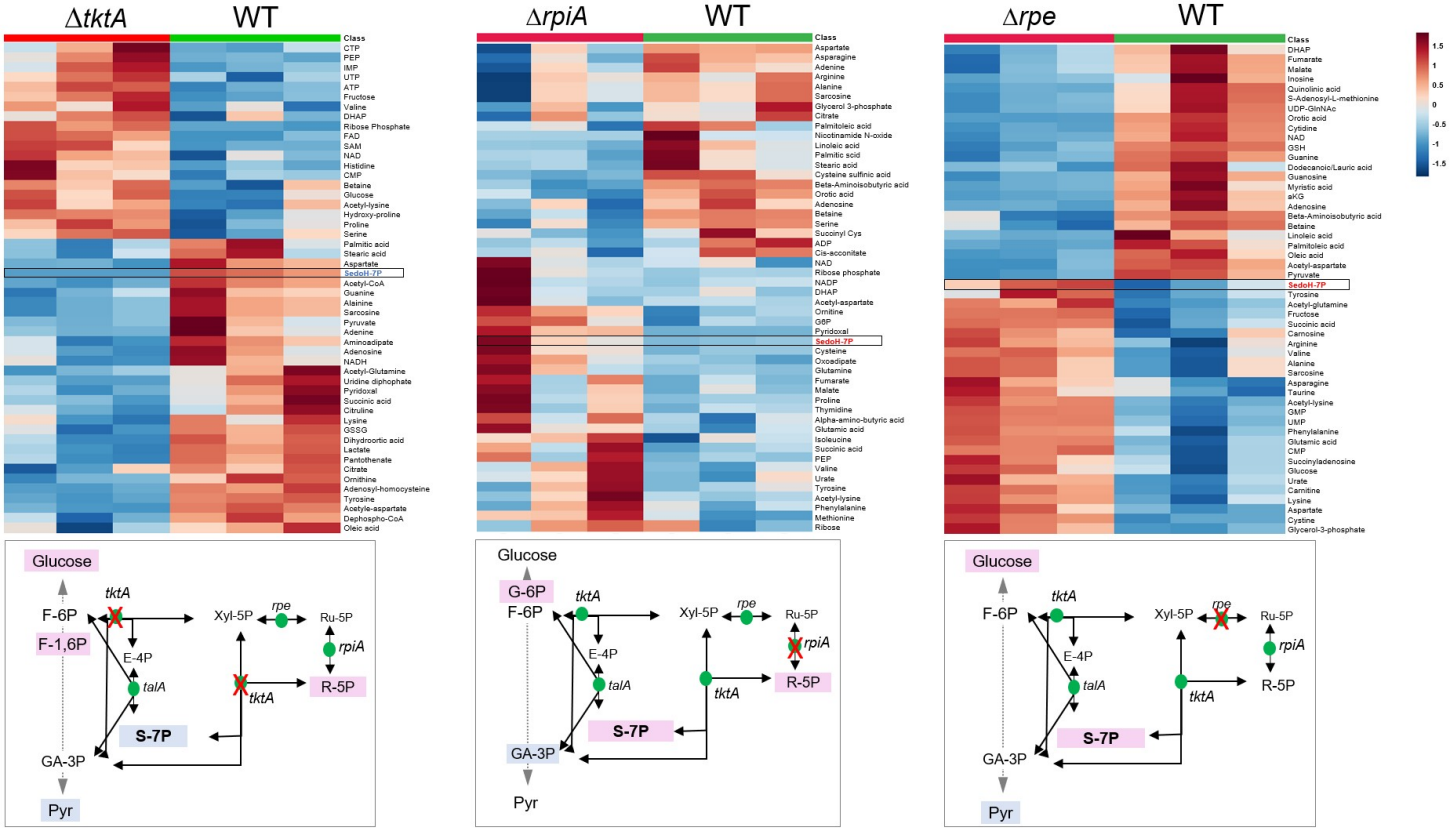
Comparison of metabolite profiles of WT and Δ*tktA*, Δ*rpe and ΔrpiA* mutants. Bacteria were cultured in TSB and collected during exponential phase of growth (at OD_600_ of 0.5). Heatmap visualization and hierarchical clustering analysis of the metabolite profiling in each mutant compared to WT *F*. *novicida*. Upper part, heatmaps showing the top 50 (Δ*tktA*, Δ*rpe*) or top 30 (Δ*rpiA*) most changing compounds. Three biological replicates, performed for each sample, are presented. Rows: metabolites; columns: samples; color key indicates metabolite relative concentration value (blue: lowest; red: highest). The arrows, to the right of each heatmap, pinpoint the metabolites related to the PPP or glycolytic pathways. Lower part, the position of the metabolites related to the PPP or glycolytic pathways is shown on a schematic depiction of the pathways.

As for proteomic analyses, bacteria were grown at 37°C in TSB to an OD_600_ of 0.5. Each strain was analyzed in three independent biological replicates (see Materials and methods). Inactivation of the *tktA* gene resulted in important changes in global metabolome (50 metabolites are presented in Fig 7). Forty metabolite levels changed significantly (23 down and 17 up). An increase of metabolites belonging to different chemical classes was recorded, including notably amino acids, nucleotides and sugars. The glucose oxidation intermediates were significantly affected with marked accumulation of glucose, the glycolytic intermediate F- 1,6P, as well as the PPP metabolite Ribose-P. It also yielded the accumulation of dihydroxyacetone phosphate (DHAP), a metabolite tightly associated to the glycolytic/gluconeogenic pathways. Indeed, DHAP is a breakdown product of fructose 1,6- bisphosphate (F-1,6P) by the enzyme fructose biphosphate aldolase (FBA) and can be converted to GA-3P by the enzyme triose phosphate isomerase.

On the contrary, *tktA* inactivation was accompanied by decreased concentrations of the PPP intermediate sedoheptulose-7P (S-7P) as well as of the glycolytic end product pyruvate. Of note, our lab recently showed that in the Gram-positive pathogen *Staphylococcus aureus*, inactivation of *tktA* also led to deregulation of whole-cell metabolism [25] and, as in *Francisella*, to accumulation of R-5P and decreased amounts of S-7P.

In *E*. *coli*, mutations in transketolase have been shown to lead to an accumulation of DHAP, a precursor of the highly toxic compound methylglyoxal (MG; [26]). To exclude a possible toxic effect of DHAP-derived MG production, we evaluated the sensitivity of the Δ*tktA* mutant to increasing concentrations of MG compared to WT *F*. *novicida* and *E*. *coli* K12 (S11 Fig). The Δ*tktA* mutant strain appeared to be even more resistant to MG treatment than the WT strain. Therefore, although this mutant produces increased amounts of MG, it is very unlikely to impact bacterial multiplication.

Consistent with what was observed with Δ*tktA*, inactivation of *rpiA* resulted in the accumulation of the glycolytic intermediate G-6P, and Ribose-P. However, unlike ΔtktA, inactivation of Δ*rpiA* resulted in increased levels of S-7P. *rpiA* inactivation was also accompanied by decreased production of the glycolytic intermediate GA-3P. *rpe* inactivation resulted in similar effect as *rpiA* with accumulation of S-7P and glucose. In addition, *rpe* inactivation resulted in decreased pyruvate. Overall Δ*rpiA* and Δ*rpe* inactivation manifested a similar metabolic phenotype that was distinct from that of the Δ*tktA* mutant.

In addition, all the mutants shared a relative decrease of long chain fatty acids (LCFAs), such as: i) palmitic and stearic acids (16 and 18 carbon, saturated LCFAs, respectively), in both Δ*tktA* and Δ*rpiA* mutants; ii) palmitoleic acid (16 carbon, monounsaturated LCFA), in Δ*rpe* and Δ*rpiA* mutants; or iii) oleic acid (18 carbon, monounsaturated FA), in Δ*tktA* and Δ*rpe* mutants. Hence, metabolic profiling of mutants inactivated in the PPP showed common traits with alterations in the relative amounts of similar glycolytic intermediates and fatty acids.

These data are also consistent with proteomic and transcriptomic analysis showing an upregulation of the whole *fad* operon and further confirm the link between the PPP and fatty acid metabolism.

### Integration of the Proteometabolomic data

**Fig 8:**
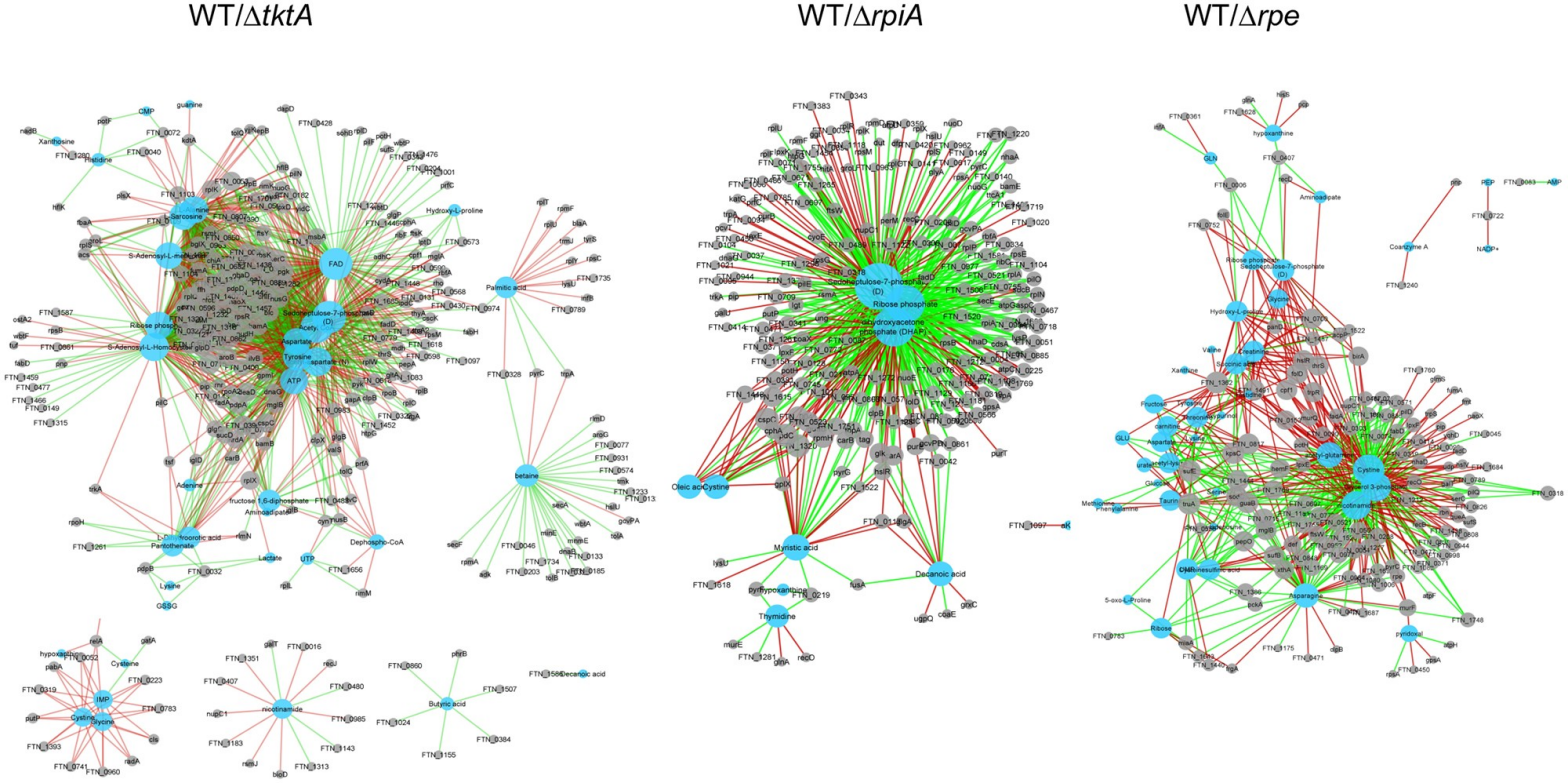
Correlation network derived from rCCA between metabolites and proteins from *Francisella*. (**A**) WT/Δ*tktA*; (**B**) WT/Δ*rpiA*; (**C**) WT/Δ*rpe*.

We anticipated that *Francisella* would reprogram its metabolism, in response to the loss of PPP enzymatic activity. Regularized Canonical Correlation Analysis (rCCA) was performed and metabolite–protein networks were identified, at a highly stringent correlation threshold of 0.95 (Fig 8 and S4 Table). Networks were performed for each pair mutant/WT, separately (Δ*tktA*/WT; Δ*rpiA/*WT and Δ*rpe*/WT). Given the linear correlation of variables used in rCCA, a correlation in the same direction was called positive and corresponded either to a decreased level of the metabolite associated with a decreased expression of the protein or to an increased level of the metabolite associated with an increased expression of the protein. Conversely, when the levels of the metabolite and the protein vary in the opposite direction, it was called a negative correlation.

For each network, three major metabolic hubs were identified, each linking one metabolite to multiple proteins (S5 Table). For the Δ*tktA/*WT network we achieved a cross-validation score (cv-score) = 0.9879 with λ1 = 0.1 and λ2 = 1.788889. The biggest hub (150 correlated proteins) was Sedopheptulose-7P (S-7P), with 80 proteins positively correlated and 70 proteins negatively correlated. The second and third largest hubs were Flavin Adenine Dinucleotide (FAD) (144 correlated proteins) and acetyl-CoA (133 correlated proteins), respectively. Of note, all three metabolites can be correlated to carbohydrate catabolic processes: S-7P as a central metabolite of the PPP; FAD as an electron carrier participating to both catabolic and anabolic reactions; and acetyl-CoA as the end product of glycolysis. For the Δ*rpiA/*WT network the cv- score was 0.9017 with λ1 = 0.0001 and λ2 = 0.9. The biggest hub (191 corelated proteins) was also Sedopheptulose-7P (S-7P), with 63 proteins positively correlated and 128 proteins negatively correlated. The second and third largest hubs were DHAP (134 corelated proteins) and the PPP metabolite ribose-P (117 corelated proteins), respectively. Of note, whereas in Δ*tktA* mutant, expression of S-7P was decreased compared to WT, in the Δ*rpiA* mutant S-7P was increased compared to WT. In this case also, all three metabolic hubs can be correlated to carbohydrate catabolic processes: S-7P and Ribose-P to the PPP; and DHAP to the glycolytic/gluconeogenic pathways. Finally, for the Δ*rpe/*WT network the cv- score was 0.9502 with λ1 = 0.0001157895 and λ2 = 0.0001894737. The biggest hub (104 corelated proteins) was cystine, with 60 proteins positively correlated and 44 proteins negatively correlated. The second and third largest hubs were the glycolytic intermediate GA- 3P (93 corelated proteins) and Nicotinamide Adenine Dinucleotide (NAD, an electron carrier like FAD, 87 corelated proteins), respectively. In contrast to the two other networks, the biggest hub (cystine) has no obvious link to carbohydrate metabolism or the PPP. However, the two other hubs, *eg*. GA-3P (a metabolite at the crossroad of glycerol metabolism, glycolysis and the PPP) and NAD, participating to both catabolic and anabolic reactions (including glycolysis and the tricarboxylic acid cycle), can be associated to these processes. The cysteine related hub was associated with 104 proteins, including approximately 80% proteins belonging to different predicted functional categories (*i*.*e*. amino acid metabolism and transport, replication and repair, translation, lipid metabolism and cell wall membrane/envelope biogenesis) and 20% proteins of unknown function. As expected, Rpe was found negatively correlated with cystine (since cystine increased and the Rpe protein decreased in the Δ*rpe*/WT network).

In both Δ*tktA*/WT and Δ*rpiA*/WT networks, S-7P was associated with a large number of proteins (150 to 191) displaying a broad spectrum of predicted biological activities (Figs 8 and S12). Notably in both cases, the largest category of proteins (except the proteins of unknown functions) was associated to the translation machinery, including the ribosomal proteins L11, L15, L17, L23, L24 of the 50S large subunit and S4, S13, S18, S30 of the 30S small subunit, for the network Δ*tktA*/WT(positively correlated since both were decreased); and L6, L11, L15, L19, L21, L24, L30 of the 50S subunit and S1, S2, S5, S17, S13 of the 30S subunit, for the network Δ*rpiA/*WT(negatively correlated since S-7P increased and the ribosomal proteins decreased). As expected, TktA was found positively correlated with S-7P (since both are decreased in the Δ*tktA*/WT network) and RpiA was found negatively correlated with S-7P (since S-7P in increased and the RpiA protein decreased in the Δ*rpiA*/WT network).

Overall, integration of our proteometabolic data highlighted links between PPP and other metabolic pathways by revealing major and/or new hubs such as S-7P and cystine. We hypothesize that such important proteometabolic hubs could greatly impact *Francisella* pathogenicity.

## Discussion

Here we showed that the pentose phosphate pathway plays an important role in the intracellular life cycle of *F*. *novicida* and constitutes a major hub for multiple metabolic pathways.

### Temporal contribution of the PPP to intracellular multiplication

Bacteria, which possess both oxidative and non-oxidative branches of PPP, are able to coordinate glycolysis/gluconeogenesis with PPP to control the production of NADPH or Ribose-5 Phosphate (R5-P), depending on environmental conditions. For example, *E*. *coli* can reroute its glycolytic flux into the oxidative branch of the PPP to achieve the immediate replenishment of NADPH for glutathione reduction upon oxidative stress [27]. On the other hand, bacteria such as *Francisella* and *Legionella*, which both lack the oxidative branch, use other, as yet unidentified pathways to produce NADPH. Of note, in *L*. *pneumophila*, the regulation of this operon is under the control of the RNA binding protein CsrA [28]. Inspection of *Francisella* genomes did not identify any potential CsrA orthologue (S2 Fig), suggesting a different mode of regulation of this locus in *Francisella* species. The fact that *gapA* expression is higher than that of *tktA* in J774.1 macrophages suggests that the glycolytic/gluconeogenic metabolic axis is probably required at a higher activity level than PPP during the bacterial intracellular life cycle. We and others have shown that gluconeogenesis was used as the main pathway for host-derived amino acids utilization as carbon and nitrogen sources [9,10,29]. It has also been shown that host cell lipolysis was required during intracellular *Francisella* replication. Hence, host-derived triglyceride stores (*i*.*e*. esters derived from glycerol and three fatty acids) may also represent a primary source of glycerol, a gluconeogenic substrate, for the bacterium in the cytosolic compartment.

Inactivation of three genes of the PPP (*tktA*, *rpe* and *rpiA*) provoked an initial intracellular growth arrest up to 10 h after infection, while at later time points, bacterial multiplication started to resume, especially with the Δ*rpe* and Δ*rpiA* mutants. These data support the idea that intracellular bacteria require the PPP (and glycolysis) during their initial phase of multiplication. However, when gluconeogenesis becomes the primary pathway for utilization of host-derived nutrients, PPP may no longer be essential (S13 Fig). To comfort this hypothesis, we designed an assay where we compared intracellular multiplication of *F*. *novicida* WT and an isogenic Δ*glpX* mutant strain (bearing a deletion of the gene encoding the unique gluconeogenic enzyme fructose 1,6-bisphosphatase, FBPase II [9], in macrophages either fed continuously with an external source of glucose (in DMEM-glucose), or fed for the first 24 hours with glucose (in DMEM-glucose); and for the next 48 hours in glucose-free medium (in DMEM-glycerol). As previously reported, in glucose-containing medium throughout the infection (in DMEM-glucose), the *glpX* mutant showed wild-type intracellular multiplication (S14A Fig). However, if glucose was replaced with glycerol after 24 h of normal growth, (S14B Fig), the number of CFUs in the Δ*glpX* mutant dropped drastically (30-fold less than WT at 48 h and more than 1 000-fold less than WT after 76 h), whereas the number of CFUs recorded with the WT strain remained similar to that in DMEM-glucose. These data strongly support the idea that when intracellular glucose stores become limited, *Francisella* depends primarily on gluconeogenesis to survive and multiply.

The interactions between *Francisella* and arthropod vectors have been shown to play a major role in its ecology and transmission to mammals [30] and *Drosophila melanogaster* has been used as a simple and easy to handle arthropod model system to study *Francisella* pathogenesis [17,21–24]. In adult flies, all the earlier studies have shown that *Francisella* was able to grows to high levels within flies and caused a lethal infection within 5 to 10 days post-infection, depending on the infectious dose used. In particular, very high bacterial levels were observed due to bacteria growing extracellularly [17,21]. Somewhat unexpectedly, we found that the three PPP mutants Δ*tktA*, Δ*rpe* and Δ*rpiA* were still virulent and able to multiply actively in infected adults *Drosophila*, suggesting that the content of these extracellular spaces of the fly is likely to fully compensate the nutritional defect provoked by PPP inactivation. Although *Francisella* has been shown to be able to multiply in hemocytes (the macrophage-like cells of *D*. *melanogaster*), *Francisella* is also able to multiply at high levels in the extracellular spaces of the head, legs, and even wings [21]. Therefore, the metabolic pathways of *Francisella* at play in the arthropod nutritional environment are likely to be very different from those required for survival in a mammalian macrophage.

### Cross-talk between PPP and other metabolic pathways

Our earlier fluxomic analyses have shown that a glycolytic flux through the PPP existed [9]. In contrast, in the presence of the gluconeogenic substrate pyruvate, compounds of the PPP were not detected, suggesting that the gluconeogenic flux from pyruvate did not involve the participation of the PPP. Carbon flux analyses performed by Eisenreich and co-workers [31] confirmed that exogenous glucose was the most efficient carbon substrate for *F*. *novicida* when growing in complex medium and suggested a possible glycolytic turnover via the PPP and/or gluconeogenesis. Both TalA and TktA enzymes act as a bridge between glycolysis and the PPP by sharing intermediate metabolites with glycolysis (F-6P and GA-3P) [32]. Somewhat unexpectedly, loss of *talA-*encoded transaldolase activity had no deleterious impact on *Francisella* intracellular fate and even improved its intracellular multiplication. Interestingly, TalA has been shown to play a key role in PPP in some bacteria but not others [33,34]. Hence, either *Francisella* possesses a functional paralog lacking significant amino acid sequence similarity with TalA, or another enzyme known to play a different role, may also be capable of compensating, under certain metabolic conditions, for the absence of transaldolase.

The fact that the three other PPP mutants (Δ*tktA*, Δ*rpe* and Δ*rpiA*) were still able to survive and replicate after 24 hours might be explained by the rescue provided by the pool of host- derived intermediates. Indeed, it has been shown in *Chlamydia* that nucleotide transport proteins (NTTs) catalyzed the import of nucleotides from the eukaryotic host into the bacterial cell and rendered de novo synthesis of these compounds dispensable [35,36]. Nucleotide utilization was also recently described in parasites [37] Microsporidia, a group of strict intracellular eukaryotic parasites which cannot make nucleotides, also use host-derived nucleotides. For this, they are equipped with multiple paralogs of the major facilitator superfamily (MFS) transporters to exploit the host nucleotide pool. *Francisella* also possesses multiple transporters [38], including phagosomal transporter (Pht) family members [39–42]. Of note, in *L*. *pneumophila*, two of these transporters, PhtC and PhtD, were proposed to contribute to protect *the bacterium* from dTMP starvation during its intracellular life cycle.

Several intracellular pathogens including, *Listeria*, *Legionella*, *Coxiella*, and *Chlamydia*, have been shown to develop a bi-modal way of using nutrients coined a bipartite metabolism [43], contributing to the temporal and spatial intracellular niche adaptation. Whereas *L*. *pneumophila* and *C*. *burnetii* species use host amino acids, and especially serine, as primary sources of carbon and energy [44,45], *L*. *monocytogenes* and *C*. *trachomatis* use host glycerol or malate, respectively [46,47], to feed their TCA cycle and *L*. *monocytogenes* uses glucose and glucose-6P primarily for biosynthesis of cell wall components and nucleotides [48].

### Novel metabolic networks at play

A recent study by Sachla and Helmann showed that the toxic metabolite 4-P-erythronate (4PE) was accidentally produced from the promiscuous reaction of GapA with erythrose-4-phosphate of the PPP [49], illustrating the multiple, and sometimes unexpected, connections between glycolysis and the PPP. Integrative Omic approaches are important to have a global view of the changes triggered upon inactivation of individual genes [50] and can allow the identification of unexpected connections between metabolic pathway. Network biology is based on the principle that biological processes are not chiefly controlled by individual molecules or by discrete, unconnected linear [51]. Here, we applied rCCA to explore correlation structures between metabolites and proteins, to explore the links between the PPP and other metabolic pathways, including glucolysis/gluconeogenesis, in *Francisella*, as well as to discovered novel metabolic crosstalks and connecting hubs.

The three major proteometabolic hubs we identified were, for the most part, predictable. Indeed, S7P, and Ribose-P link the three PPP mutants to the PPP; Ga-3P and Acetyl-coA, to the glycolysis/gluconeogenesis pathways; and DHAP to glycerol metabolism. The hubs NAD and FAD were less predictable even if *F*. *novicida* is able to produce FAD from riboflavin and the PPP metabolite ribulose-5P (according the KEGG website https://www.genome.jp/kegg-bin/show_pathway?ftn00740). The presence of NAD *de novo* synthesis is also inferred by genomic reconstruction (https://www.genome.jp/kegg-bin/show_pathway?ftn00760) and two alternative biosynthetic pathways have been experimentally demonstrated from nicotinamide and aspartate [52]. The most unexpected hub, identified by comparing Δ*rpe* to WT, was cystine. Cystine results from the dimerization of cysteine. The genome of *F*. *novicida* does not contain a gene coding for any enzyme responsible for the conversion of cystine to cysteine. We and others have previously shown that glutathione (γ-Glutamate-cysteine-glycine or GSH) constituted a major host-derived source of cysteine for intracellular *Francisella* [53,54]. It should be noted that, in addition to serve as an amino acid source for protein biosynthesis cysteine (and cystine) can also be used as sources of sulfur. This indispensable chemical element is required for the activity of many enzymes and is involved in ion and redox metabolic pathways [55]. Remarkably, three proteins involved in sulfur metabolism are correlated to cystine in the Δ*rpe*/WT pair: SufS, SufB (FTN_0851, Fe-S cluster assembly protein), and SufE. In *E*. *coli*, SufE together with the SufBCD complex, has been shown to enhance SufS cysteine desulfurase activity, as part of a sulfur transfer pathway for Fe-S cluster assembly [56]. The connection to the PPP remains to be elucidated since none of the enzymes of glycolysis or the PPP rely on Fe-S clusters.

In the Gram-positive pathogen *Staphylococcus aureus*, our laboratory has recently shown that *tktA* inactivation also led to dysregulation of the whole cell metabolism [25]. As in *Francisella*, an accumulation of R-5P and a decrease in S-7P amount were recorded in the Δ*tktA* mutant compared to wild-type *S*. *aureus*.

Of particular interest, rCCA integration of proteomic and metabolic data allowed us to identify several putative novel connections of the PPP with other pathways and notably with the Fatty acid degradation pathway (Fad) and with cystine metabolism. *Francisella* is auxotrophic for cysteine and thus absolutely relies on external sources of cysteine for growth. We found that the Fad pathway was upregulated in the three mutants Δ*tktA*, Δ*rpiA* and Δ*rpe*. qRT-PCR analyses of the *fad* locus further confirmed its upregulation in the Δ*tktA* mutant compared to WT *Francisella* (S10 Fig). In *E*. *coli*, the *fad* operon is under the negative control of the FadR repressor whose promoter binding activity has been shown to be altered upon binding of long chain unsaturated fatty acid. FadR has no ortholog in *Francisella* genomes. Hence, it is likely that the regulation of *fad* genes in *Francisella* is different from that in *E*. *coli*. Metabolomic analyses also revealed a decrease in the amounts of several long chain and middle chain fatty acids in the metabolomes of the three mutants, compatible with their increased degradation. It is tempting to suggest that this increased fatty acid degradation might be a mean for the mutant bacteria to increase the pool of acetyl-CoA (the end-product of the pathway) in order to rapidly fuel the TCA and compensate for the lack of the PPP.

It should be recalled that mutants at the *fad* locus have been repeatedly identified in previous *in vivo* and *in vitro* genetic screens of mutant libraries, for genes involved in Francisella virulence. Fully confirming the importance of fatty acid degradation in *Francisella* pathogenesis, the *fadA* gene has been identified in two genetic screens [17,57] and the *fadB* gene has been identified in multiple genetic screens [15,19,23,58].

The integration of proteomics and metabolomics data open the way to the discovery of novel metabolic crosstalks contributing and/or controlling the intracellular life cycle of *Francisella* and should apply to other intracellular bacterial pathogens, offering thus new perspectives on host-pathogen interplay.

## Materials and methods

### Ethics statement

All Materials and Methods involving animals were conducted in accordance with guidelines established by the French and European regulations for the care and use of laboratory animals (Decree 87–848, 2001–464, 2001–486 and 2001–131 and European Directive 2010/63/UE) and approved by the INSERM Ethics Committee (Authorization Number: 75–906).

### Strains and culture conditions

Strains, plasmids and primers used in this study are listed in S2 Table. All *F*. *novicida* strains used in this study are derivative from *F*. *tularensis* subsp. *novicida* U112. The *tktA* deletion mutant was constructed by allelic replacement (see below). The three transposon insertion mutants of *F*. *novicida* (*rpiA*: *FTN_1185*, *tnfn1_pw060328p07q169; rpe*: *FTN_1221*, *tnfn1_pw060419p02q104; talA*: *FTN_0781*, *tnfn1_pw060323p07q189*), were designated here Δ*rpiA*, Δ*rpe and* Δ*talA* for simplicity.

Bacteria were grown at 37°C on PolyViteX agar plates (BioMerieux), TSB, Schaedler K3, or CDM. The CDM used for *F*. *novicida* corresponds to standard CDM without threonine and valine [59]. For growth condition determination, bacterial strains were inoculated in the appropriate medium at an initial OD_600nm_ of 0.1 from an overnight culture in TSB.

#### Construction of a Δ*tktA* deletion mutant

We inactivated the gene *tktA* in *F*. *novicida* (*FTN_1333*) by allelic replacement, resulting in the deletion of the entire gene. Briefly, we generated by overlap PCR a recombinant PCR product containing the upstream region of gene *tktA* (*tktA* -UP), a kanamycin resistance cassette (*nptII* gene fused with *pGro* promoter) and the downstream region of the gene *tktA* (*tktA* -DN). Primers *tktA* upstream FW and *tktA* upstream (spl_K7) RV amplified the 700 bp region upstream of position + 1 of the *tktA* coding sequence (*tktA*-UP), primers *pGro* FW and *nptII* RV amplified the 1091 bp kanamycin resistance cassette (*nptII* gene fused with *pGro* promoter); and primers *tktA* downstream (spl_K7) FW and *tktA* downstream RV amplified the 491 bp region downstream of the position +1991 of the *tktA* gene coding sequence (*tktA*-DN). PCR reactions were realized using Phusion High-Fidelity DNA Polymerase (ThermoScientific) and PCR products were purified using NucleoSpin Gel and PCR Clean-up kit (Macherey- Nagel). The overlap PCR product was purified from agarose gel and was directly used to transform wild type *F*. *novicida* by chemical transformation [41]. Recombinant bacteria were isolated by spreading onto Chocolate agar plates containing kanamycin (10μg mL^-1^). The mutant strain was checked for loss of the wild type gene by PCR product direct sequencing (GATC-biotech) using appropriate primers.

#### Functional complementation

The plasmids pKK-*tktA*_*cp*_, pKK-*rpe*_*cp*_, and pKK-*rpiA*_*cp*_, used for complementation of the *F*. *novicida* Δ*tktA*, Δ*rpe* and Δ*rpiA*, are described below. Primers tktA [SmaI] FW and tktA [PstI] RV amplified the 236 bp region immediately upstream of *tktA* start codon and the complete *tktA* gene from U112. Primers rpe [SmaI] FW *rpe* and rpe [PstI] RV *rpe* amplified the 475 bp region immediately upstream of *rpe* start codon and the complete *rpe* gene from U112. Primers pGro FW and pGro RV amplified the 328 bp of the *pGro* promoter and primers rpiA FW (pGro)/ *rpiA [PstI] RV* amplified the 70 bp region immediately upstream of *rpiA* start codon and the complete *rpiA* gene from U112.

The PCR products were purified and SmaI/PstI restricted in presence of FastAP Thermosensitive Alkaline Phosphatase (ThermoScientific) to avoid self-ligation, and cloned into pKK214 vector after SmaI/PstI double restriction and transformed in *E*. *coli* TOP10. Recombinant plasmids pKK-*Cp* were purified and directly used for electroporation in the corresponding mutant strains [41]. Recombinant colonies were selected on PolyViteX agar plates containing tetracycline (5μg mL^-1^) and kanamycin (10μg mL^-1^).

### Proteomic analyses

Bacteria were grown at 37°C in TSB to an OD_600_ of 0.5. Bacteria were collected by centrifugation and the bacterial pellets were resuspended and lysed by sonication. Each strain was analyzed in three independent biological replicates. Protein concentration was determined by DC assay (Bio-Rad, CA) according to the manufacturer’s instructions.

*Protein digestion*: S-Trap micro spin column (Protifi, Hutington, USA) digestion was performed on 50 μg of bacterial lysates according to manufacturer’s instructions. Briefly, samples were reduced with 20mM TCEP and alkylated with 50 mM CAA (chloracetamide) for 15min at room temperature. Aqueous phosphoric acid was then added to a final concentration of 1.2% following by the addition of S-Trap binding buffer (90% aqueous methanol, 100mM TEAB, pH7.1). Mixtures were then loaded on S-Trap columns. Two extra washing steps were performed for thorough SDS elimination. Samples were digested with 2.5 μg of trypsin (Promega) at 47°C for 1h. After elution, peptides were vacuum dried and resuspended in 100μl of 10% ACN, 0.1% TFA in HPLC-grade water prior to MS analysis. For each run, 1 μL was injected in a nanoRSLC-Q Exactive PLUS (RSLC Ultimate 3000) (Thermo Scientific,Waltham MA, USA). Peptides were loaded onto a μ-precolumn (Acclaim PepMap 100 C18, cartridge, 300 μm i.d.×5 mm, 5 μm) (Thermo Scientific), and were separated on a 50 cm reversed-phase liquid chromatographic column (0.075 mm ID, Acclaim PepMap 100, C18, 2 μm) (Thermo Scientific). Chromatography solvents were (A) 0.1% formic acid in water, and (B) 80% acetonitrile, 0.08% formic acid. Peptides were eluted from the column with the following gradient 5% to 40% B (120 minutes), 40% to 80% (1 minutes). At 121 minutes, the gradient stayed at 80% for 5 minutes and, at 126minutes, it returned to 5% to re-equilibrate the column for 20 minutes before the next injection. One blank was run between each replicate to prevent sample carryover. Peptides eluting from the column were analyzed by data dependent MS/MS, using top-10 acquisition method. Peptides were fragmented using higher-energy collisional dissociation (HCD). Briefly, the instrument settings were as follows: resolution was set to 70,000 for MS scans and 17,500 for the data dependent MS/MS scans in order to increase speed. The MS AGC target was set to 3.106 counts with maximum injection time set to 200 ms, while MS/MS AGC target was set to 1.105 with maximum injection time set to 120 ms. The MS scan range was from 400 to 2000 m/z.

*NanoLC-MS/MS protein identification and quantification*: Samples were vacuum dried, and resuspended in 30 μL of 10% acetonitrile, 0.1% trifluoroacetic acid for LC-MS/MS. For each run, 1 μL was injected in a nanoRSLC-QExactive PLUS (RSLC Ultimate 3000, ThermoScientific, Waltham, MA, USA). Peptides were separated on a 50 cm reversed-phase liquid chromatographic column (Pepmap C18, Thermo Scienfitic). Chromatography solvents were (A) 0.1% formic acid in water, and (B) 80% acetonitrile, 0.08% formic acid. Peptides were eluted from the column with the following gradient of 120 min. Two blanks were run between triplicates to prevent sample carryover. Peptides eluting from the column were analyzed by data dependent MS/MS, using top-10 acquisition method. Briefly, the instrument settings were as follows: resolution was set to 70,000 for MS scans and 17,500 for the data dependent MS/MS scans in order to increase speed. The MS AGC target was set to 3Å~106 counts, while MS/MS AGC target was set to 1Å~105. The MS scan range was from 400 to 2000m/z. MS and MS/MS scans were recorded in profile mode. Dynamic exclusion was set to 30 s duration. Three replicates of each sample were analyzed by nanoLC-MS/MS.

*Data processing following nanoLC-MS/MS acquisition*: The MS files were processed with the MaxQuant software version 1.5.8.30 and searched with Andromeda search engine against the *Uniprot F*. *novicida* database (release 2016, 1 722 entries). To search parent mass and fragment ions, we set a mass deviation of 3 and 20 ppm respectively. The minimum peptide length was set to 7 amino acids and strict specificity for trypsin cleavage was required, allowing up to two missed cleavage sites. Carbamidomethylation (Cys) was set as fixed modification, whereas oxidation (Met) and N-term acetylation were set as variable modifications. The false discovery rates at the protein and peptide levels were set to 1%. Scores were calculated in MaxQuant as described previously. The reverse and common contaminants hits were removed from MaxQuant output. Proteins were quantified according to the MaxQuant label-free algorithm using LFQ intensities; protein quantification was obtained using at least 1 peptide per protein.

Statistical and bioinformatic analysis, including heatmaps, profile plots, and clustering, were performed with Perseus software (version 1.5.5.31) freely available at www.perseus-framework.org. For statistical comparison, we set two groups, WT and Δ*tktA*, each containing four biological replicates. Each sample was run in technical triplicates as well. We then filtered the data to keep only proteins with at least 3 valid values out 4 in at least one group. Next, the data were imputed to fill missing data points by creating a Gaussian distribution of random numbers with a SD of 33% relative to the SD of the measured values and 2.5 SD downshift of the mean to simulate the distribution of low signal values. We performed an T test, FDR<0.001, S0 = 1.

Hierarchical clustering of proteins that survived the test was performed in Perseus on logarithmic scaled LFQ intensities after z-score normalization of the data, using Euclidean distances.

### Metabolomic analyses

Metabolites profiling of *F*. *novicida* isolates was performed by liquid chromatography–mass spectrometry (LC-MS) as described [60]. Two independent experiments with 3 biological replicates were performed for each isolate. Briefly, bacteria were grown in the same conditions as for the proteomic analyses. Metabolic activity was blocked by immersion in liquid nitrogen for 10 sec.

Metabolites were extracted using a solvent mixture of Methanol/ACN/H_2_O (50:30:20) at -20°C. Samples were vortexed for 5 min at 4°C, and then centrifuged at 16,000 g for 15 minutes at 4°C. The supernatants were collected and analyzed by LC-MS using SeQuant ZIC-pHilic column (Millipore) for the liquid chromatography separation. The aqueous mobile- phase solvent was 20 mM ammonium carbonate plus 0.1% ammonium hydroxide solution and the organic mobile phase was acetonitrile. The metabolites were separated over a linear gradient from 80% organic to 80% aqueous phase for 15 min. The column temperature was 48°C and the flow rate was 200 μl/min. The metabolites were detected across a mass range of 75–1 000 m/z using the Q-Exactive Plus mass spectrometer (Thermo) at a resolution of 35,000 (at 200 m/z) with electrospray ionization and polarity switching mode. Lock masses were used to ensure mass accuracy below 5 ppm. The peak areas of different metabolites were determined using TraceFinder software (Thermo) using the exact mass of the singly charged ion and known retention time on the HPLC column.

Statistical analyses were performed using MetaboAnalyst 4.0 software. The algorithm for heatmap clustering was based on the Pearson distance measure for similarity and the Ward linkage method for biotype clustering. Metabolites with similar abundance patterns were positioned closer together.

### Network analysis

Aiming the metabolomics and proteomics data integration, we used the regularized canonical correlation analysis (rCCA) to build the correlation networks. The rCCA is a modification of the classical canonical correlation analysis (CCA), which is a multivariate statistical method used to assess correlations between two multivariate datasets [61]. Three biological replicates of each dataset were used (proteomics and metabolomics) and, in order to reduce the discrepancy of the scales, we combined some transformations and scaling methods, like generalized logarithm transformation [62], pareto scaling [63] or z-score. CCA aims to maximize the correlation between linear combinations of variables (canonical variates) in two datasets [64]. The rCCA analysis used is part of the mixOmics package V5.2 [65] for R (http://www.R-project.org). Regularization parameters (λ_1_ and λ_2_) were estimated using the *tune*.*rcc()* function to evaluate the cross-validation score (*cv-score*) for each point in the network, achieving the values for λ_1_ and λ_2_ that offered the highest *cv-score*. To create the canonical correlations and the canonical variates between the datasets we use the *rcc()* function and the *network()* to produce the networks. We set the correlation threshold to ≥ 0.95, this value was chosen to obtain biologically interpretable networks that were neither too sparse nor too dense [66]. After, the networks were exported to Cytoscape network visualization software V3.8 [67], using the organic layout with diameter of the node relative to the number of undirected edges. The intensity of edge colors represents the correlation values ranging from dark green (negative correlation) to dark red (positive correlation). Since hubs are network structures that could play a key role in biological networks [64,68], we focus in the three biggest hubs of each network and the direct correlations of proteins related to the *fad* operon.

The number of positive and negative correlations and the complete description of each network are reported in the S3 Table and the cytoscape (.cys) files of each network are provided in S4 Table.

### Cell cultures and cell infection experiments

J774A.1 (ATCC TIB-67™) cells were propagated in Dulbecco’s Modified Eagle’s Medium (DMEM, PAA), containing 10% fetal bovine serum (FBS, PAA) unless otherwise stated. Preparation and culture of bone marrow macrophages (BMDMs) were performed as previously described [10]. All mice were in the C57BL/6J background (Charles River, France). For CFU counting, the day before infection, approximately 2.10^5^ eukaryotic cells per well were seeded in 12-wells cell tissue plates and bacterial strains were grown overnight in 13 mL of K3 at 37°C.

Infections were realized at a multiplicity of infection (MOI) of 100 for J774.1 cells and of 200 for BMMs and incubated for one hour at 37°C in culture medium. After 3 washes with cellular culture medium, plates were incubated for 4, 10 and 24 h in fresh medium supplemented with gentamycin (10 μg mL^-1^). At each kinetic point, cells were washed 3 times with culture medium and lysed by addition of 1 mL of distilled water for 10 min at 4°C. Viable bacteria titers were determined by spreading preparations on chocolate plates. Each experiment was conducted at least twice in triplicates.

### Cytotoxicity assays

The LDH release assay was conducted as described [20]. Briefly, J774 macrophages (approximately 2.10^4^ eukaryotic cells per well) seeded in 96-well culture plates were infected in triplicate with the mutants, or wild-type *F*. *novicida* U112 at a MOI of 100 and washed at 1 h post-infection. After 10 h and 24 h, the supernatants were removed and assayed for release of LDH using the CytoTox 96 nonradioactive cytotoxicity assay (Promega, Madison, WI). Cytotoxicity was determined by calculating LDH release as a percentage of the maximal amount released from macrophages lysed with detergent. Experiment was conducted 3 times.

The trypan blue exclusion assay was conducted as described [69]. Briefly, J774 macrophages (approximately 5.10^5^ eukaryotic cells per well) seeded in 6-well culture plates were infected in triplicate with the mutants, or wild-type *F*. *novicida* U112 at a MOI of 100 and washed at 1 h post-infection. After 10 h and 24 h, cells were resuspended in an equivalent volume of 0.4% trypan blue solution (Sigma Aldrich) to facilitate counting of live (trypan blue negative) or dead (trypan blue positive) cells using an automated cell counter (BioRad). Cell death was determined as the percentage of dead cells (in blue) in one well. 2 measurements were made per well. Each experiment was conducted three times.

### Fly infections

To monitor virulence, 20 adult male *Drosophila* were infected per strain (WT *F*. *novicida*, Δ*fpi* or PPP mutant), by being pricked with a glass needle dipped into a freshly isolated bacterial colony on chocolate agar PolyViteX plates (BioMerieux).

The flies were incubated at 29°C and transferred to fresh food daily. Living animals were counted once a day for 10 days, and the results were recorded as a percentage of the number of living animals relative to the number of flies recorded one day post-pricking. Flies pricked by a clean needle were considered as control of the experiment. Two independent replicates were carried out. The Gehan–Breslow–Wilcoxon test was used to compare the killing efficiency of the different mutants to that of WT *F*. *novicida*.

To monitor bacterial multiplication, 50 adult male *Drosophila* were infected per strain (WT *F*. *novicida*, Δ*fpi* or PPP mutant), by being pricked with a glass needle dipped into a freshly isolated bacterial colony on chocolate agar PolyViteX plates. Flies were incubated at 29°C and transferred daily to fresh food containing 40 μg mL^-1^ tetracycline (Tet, all bacterial strains are tetracycline resistant because they contain the TetR plasmid pKK214-GFP). Bacterial multiplication was assessed over an 8-day period for the Δ*fpi* strain (counts were performed every 4 days); over a 6-day period for the Δ*rpiA*, Δ*rpe*, and Δ*tktA* strains (counts were performed every other day); and over a 4-day period for the WT and Δ*talA* strains (counts were performed every other day). For each strain, at the specified time point, 8 flies were homogenized in 500 μl of PBS solution before being placed on chocolate PolyVitex plates (containing 5 μg mL^-1^ Tet). Bacterial quantifications were repeated three times, with n = 8 samples per repeat. Results are presented as means and standard deviation of three experiments.

### Transcriptional analysis

#### Isolation of total RNA and reverse transcription

For transcriptional analyses of bacteria grown in CDM supplemented either with glucose or glycerol, cultures were centrifuged for 2 min in a microcentrifuge at room temperature and the pellet was quickly resuspended in Trizol solution (Invitrogen, Carlsbad, CA, USA). For transcriptional analyses of bacteria in infected cells, J774.1 macrophages grown in standard DMEM-glucose medium were infected with wild-type *F*. *novicida* (WT) strain for 24 h. Cells were then collected by scratching, centrifuged at maximum speed in a microcentrifuge at room temperature and the pellet was quickly resuspended in Trizol solution. Samples were either processed immediately or frozen in liquid nitrogen and stored at −80°C. Samples were treated with chloroform and the aqueous phase was used in the Monarch RNA Cleanup Kit (New England Biolabs Inc).

#### Quantitative real-time RT-PCR

WT *F*. *novicida* and mutant strains were grown overnight at 37°C. Then, samples were harvested and RNA was isolated and reverse transcripted in cDNA following protocol manufacturer (LunaScript RT SuperMix Kit, New England Biolabs Inc). The 20 μL reaction consisted in 4 μL of RT Mix completed with 16 μL of water with 100 ng of RNA. qPCR was performed according to manufacturer’s protocol on Applied Biosystems-ABI PRISM 7700 instrument (Applied Biosystems). We used Luna Universal qPCR Master Mix (New England Biolabs, Inc), following protocol manufacturer, by adding 1 μL from RT mix. Transcript levels were analyzed using a 7900HT Fast Real-Time PCR System (Applied Biosystems) according to the standard settings of the system software. The thermal cycling conditions were: 50°C for 2 min, 95°C for 10 min, followed by 40 cycles of 95°C for 15 s and 60°C for 1 min. We used the “Relative Standard Curve Method” to analyze qRT-PCR data. The amounts of each transcript were normalized to helicase rates (*FTN_1594*). The primers used in this study are listed in S2 Table.

### *In silico* analyses

For synteny prediction, we used the Kyoto Encyclopedia of Genes and Genomes (KEGG) Sequence Similarity Database (SSDB) to search conserved gene clusters containing homologs of *FTN_1333* using default parameters (gap size 0, threshold 100).

The SSDB database (Release 94.0, last accessed on 14th April 2020) contains SSEARCH computation results (based on the Smith-Waterman similarity score) for all pairwise genome comparisons of the KEGG database (10.1093/nar/gkw1092).

The Microbial Genomic context Viewer MGcV allowing the coloring of the genes by Clusters of orthologous gene (COG) category retrieved from NCBI RefSeq was used for visualization (10.1186/1471-2164-14-209).

### Confocal experiments

The day before infection, approximately 2.10^4^ eukaryotic cells per well were seeded in 8-wells chambered coverslip (Ibidi) and bacterial strains were grown overnight in 13 mL of K3 at 37°C. J774.1 macrophages were infected (MOI of 1 000) with wild-type *F*. *novicida* U112 (WT), the pentose phosphate pathway mutants (Δ*tktA*, Δ*rpe*, Δ*rpiA*, Δ*talA*), or an isogenic strain deleted for the “Francisella Pathogenicity Island” (ΔFPI) in standard DMEM (DMEM-glucose) for 30 min at 37°C. All the bacterial strain carried a GFP-carried-plasmid. Cells were then washed three times with PBS and maintained in fresh DMEM supplemented with gentamycin (10 μg mL^-1^) until the end of the experiment. Three kinetic points (1 h, 10 h and 24h) were sampled. For each point cells were washed with 1X PBS, fixed 15 min with 4% Paraformaldehyde, and incubated 10 min in 50 mM NH_4_Cl in 1X PBS to quench free aldehydes. Cells were then blocked and permeabilized with PBS containing 0.1% saponin and 1% bovin serum albumin for 10 min at room temperature. Cells were then incubated for 30 min with anti-LAMP-1 rabbit polyclonal antibody (1/100 final dilution, ABCAM) After washing, cells were incubated for 30 min with Alexa546 conjugated donkey anti rabbit secondary antibodies (1/400e, AbCam). After washing, DAPI was added (1/10 000e) for 1 min and cells were washed and maintained in PBS. Cells were examined using an X63 oil- immersion objective on a Leica SP8 gSTED confocal microscope. Co-localization tests were quantified by using Image J software; and mean numbers were calculated on more than 100 cells for each condition. Percentage of infected cells was quantified by using ImageJ software. At least 1 000 cells per condition were counted. The number of GFP-positive spots per infected cell was quantified by using the Icy Software. We analyzed at least 100 infected cells for each condition. Confocal microscopy analyses were performed at the Cell Imaging Facility (Faculté de Médecine Necker Enfants-Malades).

### Time lapse video microscopy

#### Constructions of GFP-expressing strains

Plasmid pKK-pGro-GFP [70] was introduced by chemical transformation into wild-type *F*. *novicida* (designated WT-GFP) and in the Δ*tktA*, Δ*rpiA*, Δ*rpe* and Δ*talA* mutants, to generate strains that constitutively expressing GFP.

#### Time-lapse microscopy

The three GFP-expressing strains were imaged using the IncuCyte technology. J774.1_red_ cells were grown to confluence in 96-well cell tissue plates and were infected with GFP-expressing wild-type and mutant bacteria, at a MOI of 100. Plates were then incubated for 1 h at 37°C in culture medium. After 3 washes with cellular culture medium, plates were incubated at 5% CO2 and 37°C for 48 h in fresh medium with 5% of SVF supplemented with gentamycin (10 g mL^-1^). Bacterial multiplication was monitored in the fully automated microscope Incucyte S3 (Essen BioScience). Images were taken every 20 min with the 20X objective. Green and red fluorescence images were obtained every 20 min with an acquisition time of 400 milliseconds (ms) and 200 ms respectively. Time-lapse videos (from which images were extracted) were generated by using Incucyte S3 and imageJ softwares.

#### Data analyses

Raw images of the phase (representing the cells) as well as green fluorescence representing the GFP-labelled bacteria) were extracted for each time point. We used the open source program IlastiK v1.3.3post3 to train the machine to recognize the cells from the phase images. The same method was used to define and recognize the bacteria via the green fluorescence images. For each category (cells or bacteria), a mask was extracted from the analysis. A segmentation by size filter was carried out, using the Fiji program, on the "cells" mask in order to exclude the cellular debris. Then, the cells and bacterial masks were superimposed. This procedure allowed to: i) measure the total number of cells, at each time point, and ii) for each cell, at each time point, the presence of a GFP signal (bacteria) and the GFP area were measured (S8 Fig). The assay was performed on 8 wells for each condition.

### Statistics

*In vitro* experiments were at least repeated twice and in triplicates. Data were analyzed using GraphPad Prism software. Tests are specified in each legend. In figures, all the results correspond to mean ± SEM.

## Supporting information

S1 Fig The major steps of the PPP and the *tktA-gapA* region. (**A**) Schematic depiction of the major steps of the PPP and glycolytic/gluconeogenic pathways. (**B**) Promoter prediction upstream of ***gapA***. In bold blue characters, the predicted σ70 binding site. In green italics, the intergenic region between *tktA* and *gapA* genes. In bold, the ATG initiation codon of *gapA* gene (start).
(TIF)

S2 Fig *tktA* gene cluster synteny analysis. (**A**) *tktA* gene cluster synteny analysis within representative *Francisella* genomes. Comparative context map of *FTN_1333* homologs obtained via KEGG Sequence Similarity Database (SSDB) search is visualized using MGcV. Genes belonging to the same Clusters of orthologous gene (COG) groups are depicted in the same color. COG color key: light green: Carbohydrate transport and metabolism; dark green: Lipid transport and metabolism; red: Translation, ribosomal structure and biogenesis; grey: Hypothetical proteins. (**B**) *tktA* gene cluster synteny analysis within representative genomes of selected plant and human pathogens. Comparative context map of *FTN_1333* homologs obtained via KEGG Sequence Similarity Database (SSDB) search is visualized using MGcV. Genes belonging to the same Clusters of orthologous gene (COG) groups are depicted in the same color. COG color key: light green: Carbohydrate transport and metabolism; dark green: Lipid transport and metabolism; red: Translation, ribosomal structure and biogenesis; grey: Hypothetical proteins; white: unknown function; yellow: Energy production and conversion; brown: Inorganic ion transport and metabolism; orange: transcription; blue: Nucleotide transport and metabolism.
(TIF)

S3 Fig Growth in CDM and intracellular multiplication of complemented strains. Bacterial growth was monitored in CDM supplemented with glucose at a final concentration of 25 mM. Stationary-phase bacterial cultures of wild-type *F*. *novicida* (WT) and **(A)** Δ*rpiA*, Δ*rpiA* cpΔ **(B)** Δ*rpE*, Δ*rpE cp*, **(C)** *tktA*, Δ*tktA*, *cp*, mutants were diluted to a final OD_600nm_ of 0.1, in 20 mL broth. Every hour, the OD_600nm_ of the culture was measured, during a 24 h-period. Kinetics of intracellular multiplication of the control strains and **(D)** Δ*rpiA*, Δ*rpiA cp*, **(E)** Δ*rpE*, Δ*rpE cp*, **(F)** *tktA*, Δ*tktA cp* were monitored in J774.1 macrophages over a 24 h-period in DMEM supplemented with glucose. **, p <0.01; ***, p <0.001 (compared to WT strain; as determined by two-way ANOVA test).
(TIF)

S4 Fig Percentage of entry. The percentage of entry is the number of bacteria after one hour of infection compared to the number of bacteria in the inoculum. The percentages of entry of the different mutants were then related to the percentage of entry of the wild type strain. Experiment was carried in triplicate. **, p <0.01 (compared to WT strain; as determined by two-way ANOVA test).
(TIF)

S5 Fig Cytotoxicity. We quantified cytotoxicity at 10 h and 24 h after infection in J774.1 macrophages (MOI of 100). (**A**) Cytopathogenicity of J774.1 cells was assayed by release of LDH, using the CytoTox 96 nonradioactive cytotoxicity assay and expressed as % of release relative to that observed after lysis of the cells. **, p <0.01; ***p<0,0001 (compared to non infected condition; determined by ANOVA one-way test). (**B**) Cytopathogenicity of J774.1 cells was assayed by blue trypan assay. The number of live (unstained) and dead (blue) cells was counted using an automated cell counter.
(TIF)

S6 Fig Intracellular multiplication in bone marrow macrophages. Kinetics of intracellular multiplication of the mutants was monitored in murine bone marrow macrophages over a 24 h-period in DMEM supplemented with glucose and 10% of FBS, and compared to that in the wild-type *F*. *novicida* (WT). A Δ*fpi* mutant strain was used as a negative control. *, p <0.05; **, p <0.01; (compared to WT strain; as determined by two-way ANOVA test).
(TIF)

S7 Fig Screenshots of time lapse video microscopy. Pictures of GFP-expressing wild-type *F*. *novicida* (WT), Δ*talA*, Δ*rpiA*, Δ*rpe*Δ, *tktA* or Δ*fpi* strains, were taken one hour after infection of J774.1_red_ cells and gentamycin washing (0 h), 10 h and 24 h after intracellular survival. Bar = 50 μm.
(TIF)

S8 Fig Bioinformatic analysis of time-lapse images. (**A**) The opensource program IlastiK v1.3.3post3 recognize the cells from the phase images and the bacteria from the green fluorescence images, respectively. (**B**) A segmentation by size filter was carried out, using the Fiji program, on the "cells" mask in order to exclude the cellular debris. Then, the cells and bacterial masks were superimposed.
(TIF)

S9 Fig Virulence in the adult fly model. (**A**) Survival curves of adult male flies infected by WT strain, Δ*tkt*, Δ*rpiA*, Δ*rpe*, Δ*talA*, Δfpi mutant at 29°C. Results were analysed by Gehan–Breslow–Wilcoxon test (compared to WT strain; **p < 0.01).(**B**) CFU counts in infected flies. One point corresponds to the means of 3 groups of 8 flies that were lysed and spread on plate. Bacterial multiplication was assessed over an 8-day period for the Δ*fpi* strain; over a 6-day period for the Δ*rpiA*, Δ*rpe*, and Δ*tktA* strains; and over a 4-day period for the WT and Δ*talA* strains. 0 day post-infection corresponds to 2h after infection. Results are presented as standard deviation of three experiments. *, p <0.05; (compared to WT strain; as determined by two-way ANOVA test).
(TIF)

S10 Fig The Fatty acid degradation locus Fad. (**A**) Schematic organization of the *fad* operon. (**B**) qRT-PCR analysis. (**C**) Schematic representation of the metabolic pathway leading to acetyl-CoA production. (**D**) Proteometabolic analysis of *fad*-encoded proteins and their relationship to metabolite changes in Δ*tkt*, Δ*rpiA* and Δ*rpe* mutants.
(TIF)

S11 Fig Growth of Δ*tktA* mutant in presence of Methylglyoxal. WT and Δ*tktA* growth were monitored in TSB supplemented or not with 1mM, 5mM or 10 mM of MG. Stationary-phase bacterial cultures of wild-type *F*. *novicida* (WT) and Δ*tktA*, mutants were diluted to a final OD_600nm_ of 0.1, in 20 mL broth. Every hour, the OD_600nm_ of the culture was measured, during an 8 h-period.
(TIF)

S12 Fig Families of proteins identified with the 3 major metabolic hub of the Δ*tkt*, Δ*rpiA*, Δ*rpe* mutants. (TIF)

S13 Fig Possible interplay between PPP and glycolysis/gluconeogenesis during macrophage infection. Left panel (early), during the first 24 h of the intracellular life cycle, when glucose is still available in replete condition, glycolysis (blue arrow) and the PPP are “On”. Right panel (late), at later time points (24–48 h), when glucose becomes limiting, gluconeogenesis prevails (pink arrow) and the PPP is no longer used “Off”. Different alternative carbons sources are then used to feed the gluconeogenic pathway such as: amino acids (aas); fatty acids (long chain with aliphatic tails of 13 to 21 carbons; medium chain with aliphatic tails of 6–12 carbons; and short chain fatty acids with aliphatic tails of 2–6 carbons), designated LCFA, MCFA, SCFA respectively; or triglycerides (TG).
(TIF)

S14 Fig Intracellular multiplication of Δ*glpx* mutant. Kinetics of intracellular multiplication of the mutants was monitored in J774.1 macrophages over a 72 h-period in DMEM supplemented with (**A**) glucose or (**B**) substitution of glucose with glycerol after 24h of infection and compared to that in the wild-type *F*. *novicida* (WT). Grey arrow: change to glycerol. ***, P <0.001 (compared to WT strain; as determined by two-way ANOVA test).
(TIF)

S1 Table List of FTN_1333 homologs with proteins predicted to be in a gene cluster according to KEGG SSDB. (XLS)

S2 Table Strains, plasmids and primers used in this study. (DOCX)

S3 Table List of 135 differentially expressed in the 3 mutants Δ*tktA*, Δ*rpiA* and Δ*rpe*, compared to wild-type *F*. *novicida* (WT). (XLSX)

S4 Table Cytoscape files. Each file has the main network, the three biggest hubs, and the *fad* locus.
(ZIP)

S5 Table rCCA summary lists the number of proteins associated with the three major hubs of each pair Mutant/WT. (DOCX)

S1 Movie J774.1_red_ macrophages were infected in DMEM-High glucose at an MOI of 100 with GFP-expressing wild-type *F*. *novicida*. Images were taken every 20 minutes during a 48 h-period. Red: cell nuclei. Green: GFP expressing bacteria.
(AVI)

S2 Movie J774.1_red_ macrophages were infected in DMEM-High glucose at an MOI of 100 with GFP-expressing Δ*rpiA* mutant. Images were taken every 20 minutes during a 48 h-period. Red: cell nuclei. Green: GFP expressing bacteria.
(AVI)

S3 Movie J774.1_red_ macrophages were infected in DMEM-High glucose at an MOI of 100 with GFP-expressing Δ*rpe* mutant. Images were taken every 20 minutes during a 48 h-period. Red: cell nuclei. Green: GFP expressing bacteria.
(AVI)

S4 Movie J774.1_red_ macrophages were infected in DMEM-High glucose at an MOI of 100 with GFP-expressing Δ*tktA* mutant. Images were taken every 20 minutes during a 48 h-period. Red: cell nuclei. Green: GFP expressing bacteria.
(AVI)

S5 Movie J774.1_red_ macrophages were infected in DMEM-High glucose at an MOI of 100 with GFP-expressing Δ*talA* mutant. Images were taken every 20 minutes during a 48 h-period. Red: cell nuclei. Green: GFP expressing bacteria.
(AVI)
