# Supplementary figures and images for: The pentose phosphate pathway constitutes a major metabolic hub in pathogenic Francisella
Source: PLoS Pathog. 2021 Aug 2;17(8):e1009326. doi: 10.1371/journal.ppat.1009326 (PMC8360588; doi:10.1371/journal.ppat.1009326)

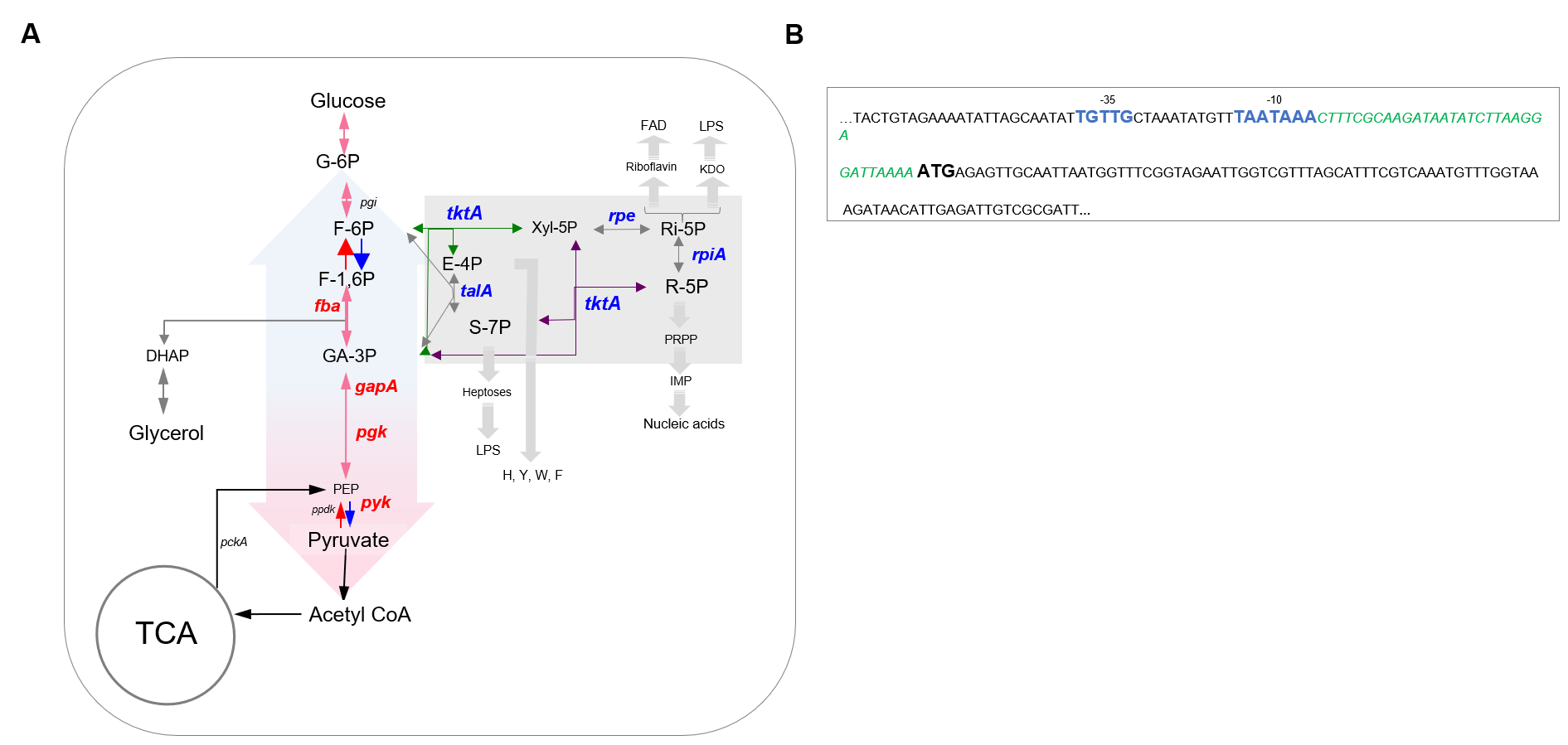

Supplement: S1 Fig — (A) Schematic depiction of the major steps of the PPP and glycolytic/gluconeogenic pathways. (B) Promoter prediction upstream of gapA. In bold blue characters, the predicted σ70 binding site. In green italics, the intergenic region between tktA and gapA genes. In bold, the ATG initiation codon of gapA gene (start). (TIF) [file ppat.1009326.s001.tif]

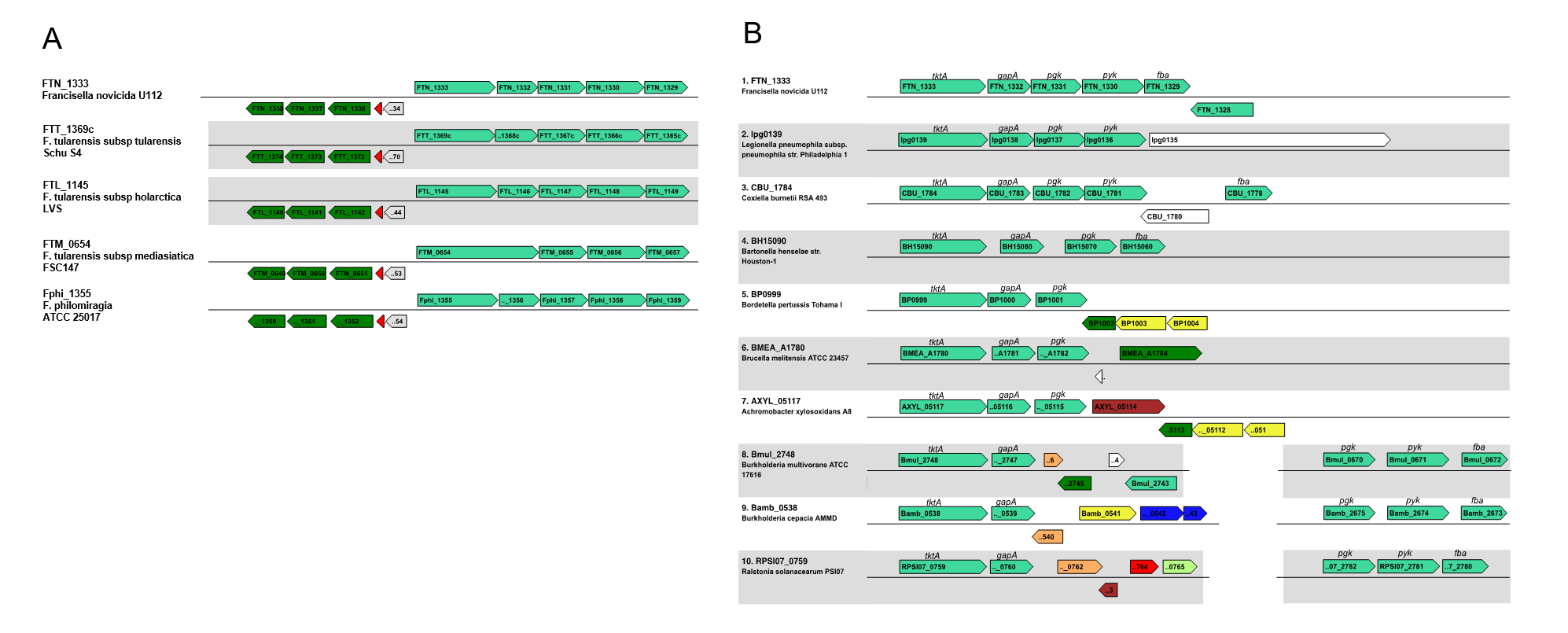

Supplement: S2 Fig — (A) tktA gene cluster synteny analysis within representative Francisella genomes. Comparative context map of FTN_1333 homologs obtained via KEGG Sequence Similarity Database (SSDB) search is visualized using MGcV. Genes belonging to the same Clusters of orthologous gene (COG) groups are depicted in the same color. COG color key: light green: Carbohydrate transport and metabolism; dark green: Lipid transport and metabolism; red: Translation, ribosomal structure and biogenesis; grey: Hypothetical proteins. (B) tktA gene cluster synteny analysis within representative genomes of selected plant and human pathogens. Comparative context map of FTN_1333 homologs obtained via KEGG Sequence Similarity Database (SSDB) search is visualized using MGcV. Genes belonging to the same Clusters of orthologous gene (COG) groups are depicted in the same color. COG color key: light green: Carbohydrate transport and metabolism; dark green: Lipid transport and metabolism; red: Translation, ribosomal structure and biogenesis; grey: Hypothetical proteins; white: unknown function; yellow: Energy production and conversion; brown: Inorganic ion transport and metabolism; orange: transcription; blue: Nucleotide transport and metabolism. (TIF) [file ppat.1009326.s002.tif]

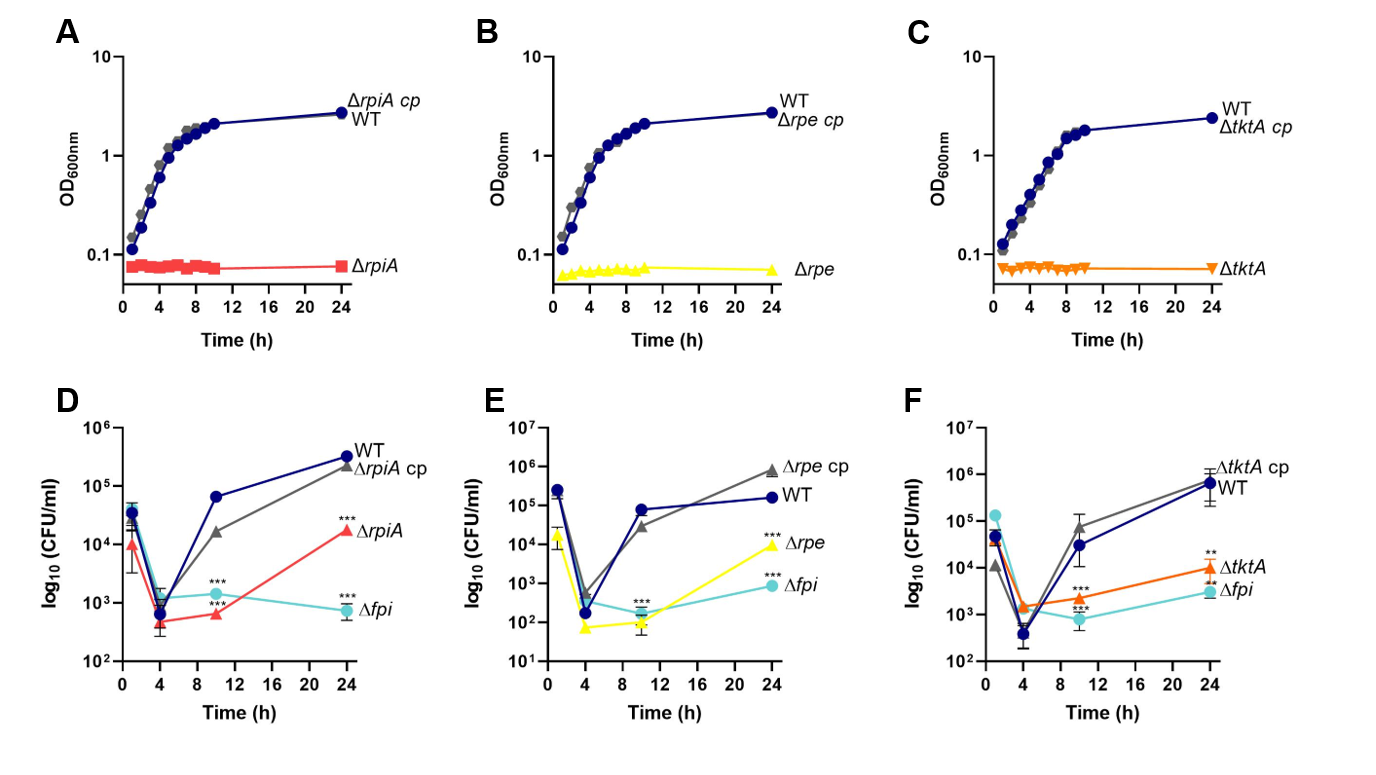

Supplement: S3 Fig — Bacterial growth was monitored in CDM supplemented with glucose at a final concentration of 25 mM. Stationary-phase bacterial cultures of wild-type F. novicida (WT) and (A) ΔrpiA, ΔrpiA cpΔ (B) ΔrpE, ΔrpE cp, (C) tktA, ΔtktA, cp, mutants were diluted to a final OD600nm of 0.1, in 20 mL broth. Every hour, the OD600nm of the culture was measured, during a 24 h-period. Kinetics of intracellular multiplication of the control strains and (D) ΔrpiA, ΔrpiA cp, (E) ΔrpE, ΔrpE cp, (F) tktA, ΔtktA cp were monitored in J774.1 macrophages over a 24 h-period in DMEM supplemented with glucose. **, p <0.01; ***, p <0.001 (compared to WT strain; as determined by two-way ANOVA test). (TIF) [file ppat.1009326.s003.tif]

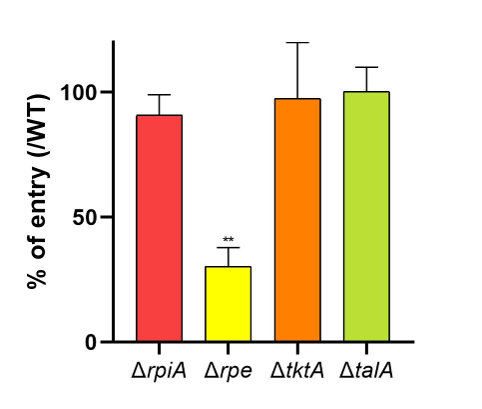

Supplement: S4 Fig — The percentage of entry is the number of bacteria after one hour of infection compared to the number of bacteria in the inoculum. The percentages of entry of the different mutants were then related to the percentage of entry of the wild type strain. Experiment was carried in triplicate. **, p <0.01 (compared to WT strain; as determined by two-way ANOVA test). (TIF) [file ppat.1009326.s004.tif]

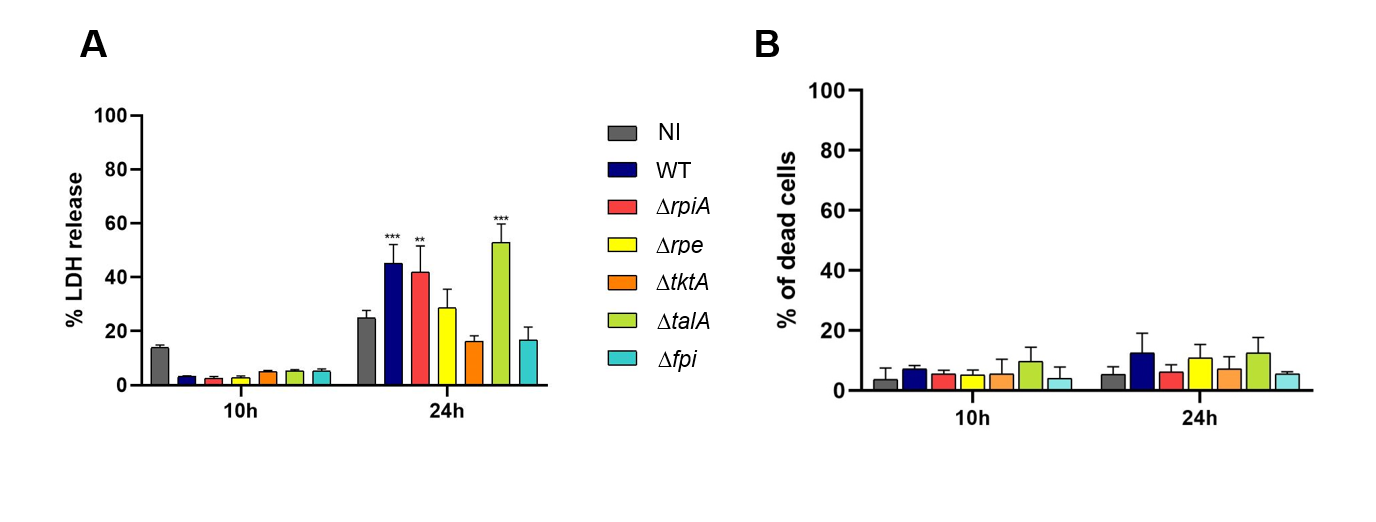

Supplement: S5 Fig — We quantified cytotoxicity at 10 h and 24 h after infection in J774.1 macrophages (MOI of 100). (A) Cytopathogenicity of J774.1 cells was assayed by release of LDH, using the CytoTox 96 nonradioactive cytotoxicity assay and expressed as % of release relative to that observed after lysis of the cells. **, p <0.01; ***p<0,0001 (compared to non infected condition; determined by ANOVA one-way test). (B) Cytopathogenicity of J774.1 cells was assayed by blue trypan assay. The number of live (unstained) and dead (blue) cells was counted using an automated cell counter. (TIF) [file ppat.1009326.s005.tif]

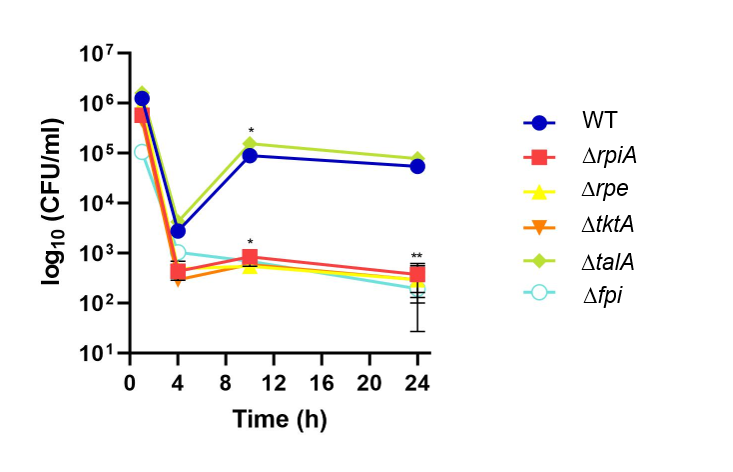

Supplement: S6 Fig — Kinetics of intracellular multiplication of the mutants was monitored in murine bone marrow macrophages over a 24 h-period in DMEM supplemented with glucose and 10% of FBS, and compared to that in the wild-type F. novicida (WT). A Δfpi mutant strain was used as a negative control. *, p <0.05; **, p <0.01; (compared to WT strain; as determined by two-way ANOVA test). (TIF) [file ppat.1009326.s006.tif]

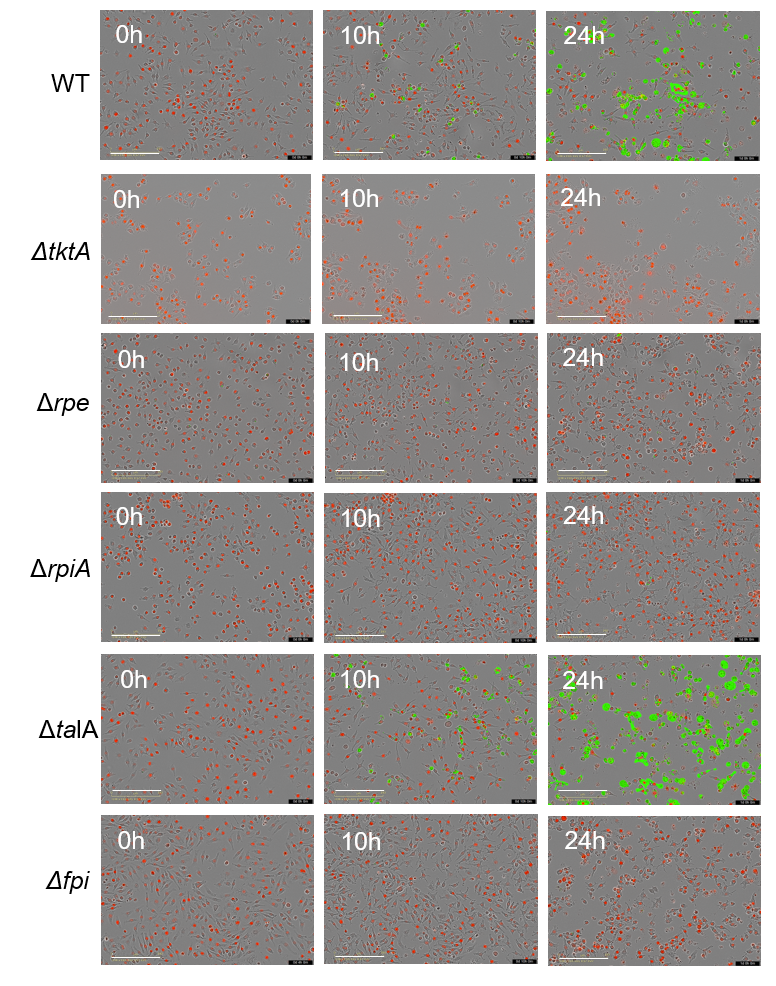

Supplement: S7 Fig — Pictures of GFP-expressing wild-type F. novicida (WT), ΔtalA, ΔrpiA, ΔrpeΔ, tktA or Δfpi strains, were taken one hour after infection of J774.1red cells and gentamycin washing (0 h), 10 h and 24 h after intracellular survival. Bar = 50 μm. (TIF) [file ppat.1009326.s007.tif]

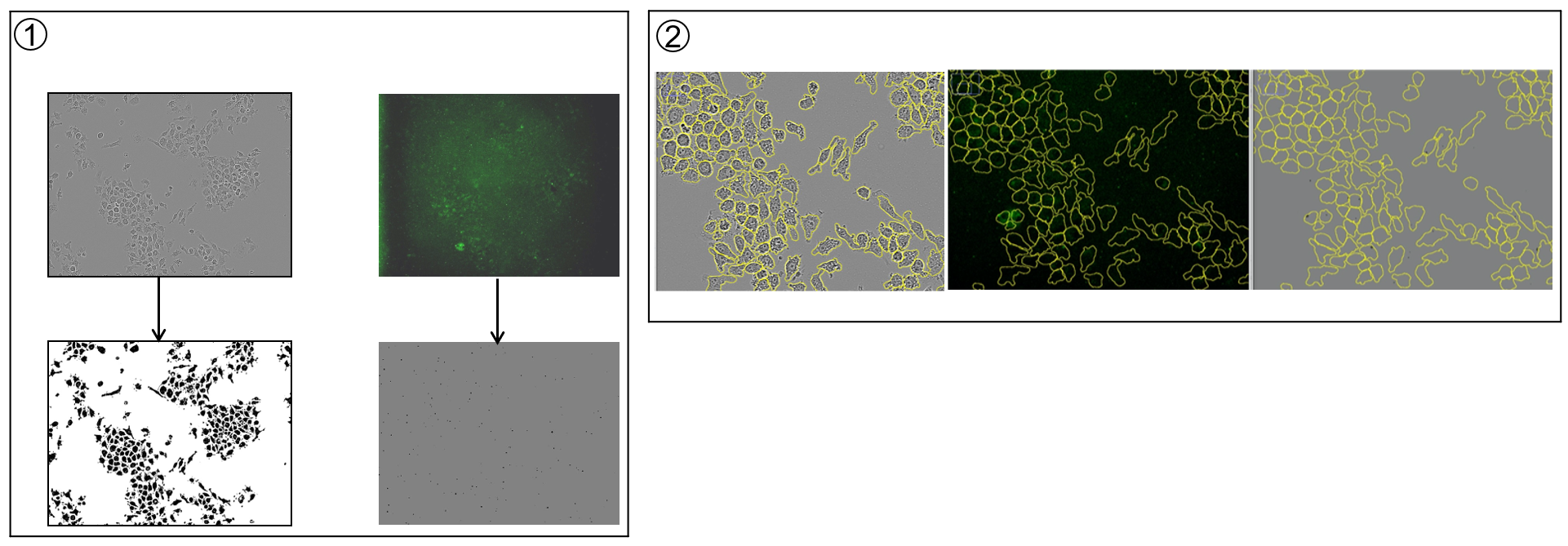

Supplement: S8 Fig — (A) The opensource program IlastiK v1.3.3post3 recognize the cells from the phase images and the bacteria from the green fluorescence images, respectively. (B) A segmentation by size filter was carried out, using the Fiji program, on the "cells" mask in order to exclude the cellular debris. Then, the cells and bacterial masks were superimposed. (TIF) [file ppat.1009326.s008.tif]

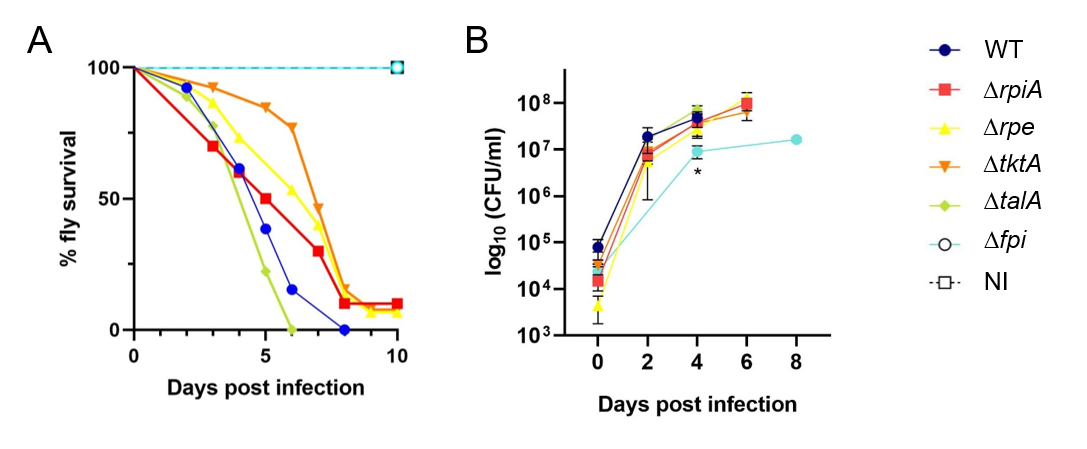

Supplement: S9 Fig — (A) Survival curves of adult male flies infected by WT strain, Δtkt, ΔrpiA, Δrpe, ΔtalA, Δfpi mutant at 29°C. Results were analysed by Gehan–Breslow–Wilcoxon test (compared to WT strain; **p < 0.01).(B) CFU counts in infected flies. One point corresponds to the means of 3 groups of 8 flies that were lysed and spread on plate. Bacterial multiplication was assessed over an 8-day period for the Δfpi strain; over a 6-day period for the ΔrpiA, Δrpe, and ΔtktA strains; and over a 4-day period for the WT and ΔtalA strains. 0 day post-infection corresponds to 2h after infection. Results are presented as standard deviation of three experiments. *, p <0.05; (compared to WT strain; as determined by two-way ANOVA test). (TIF) [file ppat.1009326.s009.tif]

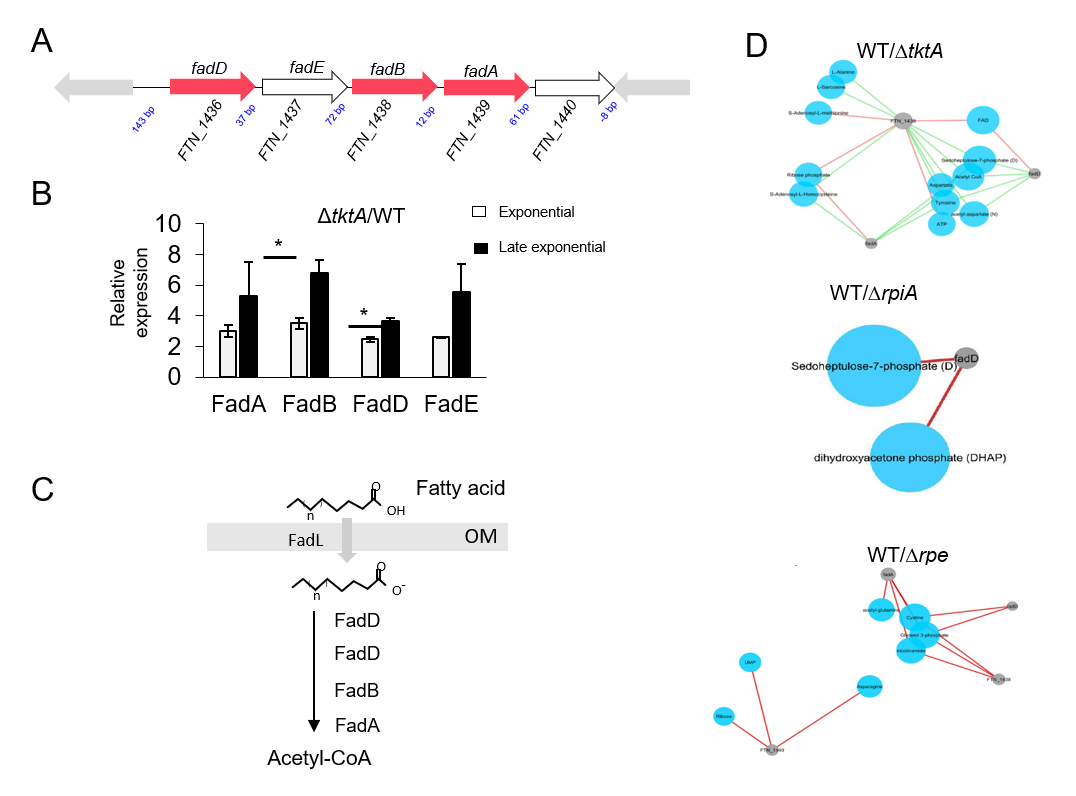

Supplement: S10 Fig — (A) Schematic organization of the fad operon. (B) qRT-PCR analysis. (C) Schematic representation of the metabolic pathway leading to acetyl-CoA production. (D) Proteometabolic analysis of fad-encoded proteins and their relationship to metabolite changes in Δtkt, ΔrpiA and Δrpe mutants. (TIF) [file ppat.1009326.s010.tif]

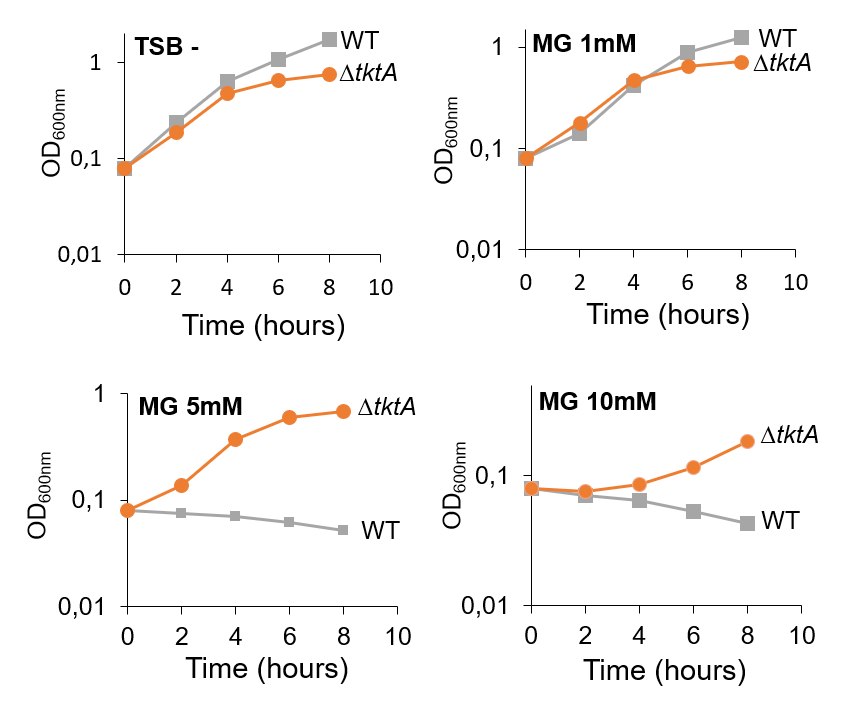

Supplement: S11 Fig — WT and ΔtktA growth were monitored in TSB supplemented or not with 1mM, 5mM or 10 mM of MG. Stationary-phase bacterial cultures of wild-type F. novicida (WT) and ΔtktA, mutants were diluted to a final OD600nm of 0.1, in 20 mL broth. Every hour, the OD600nm of the culture was measured, during an 8 h-period. (TIF) [file ppat.1009326.s011.tif]

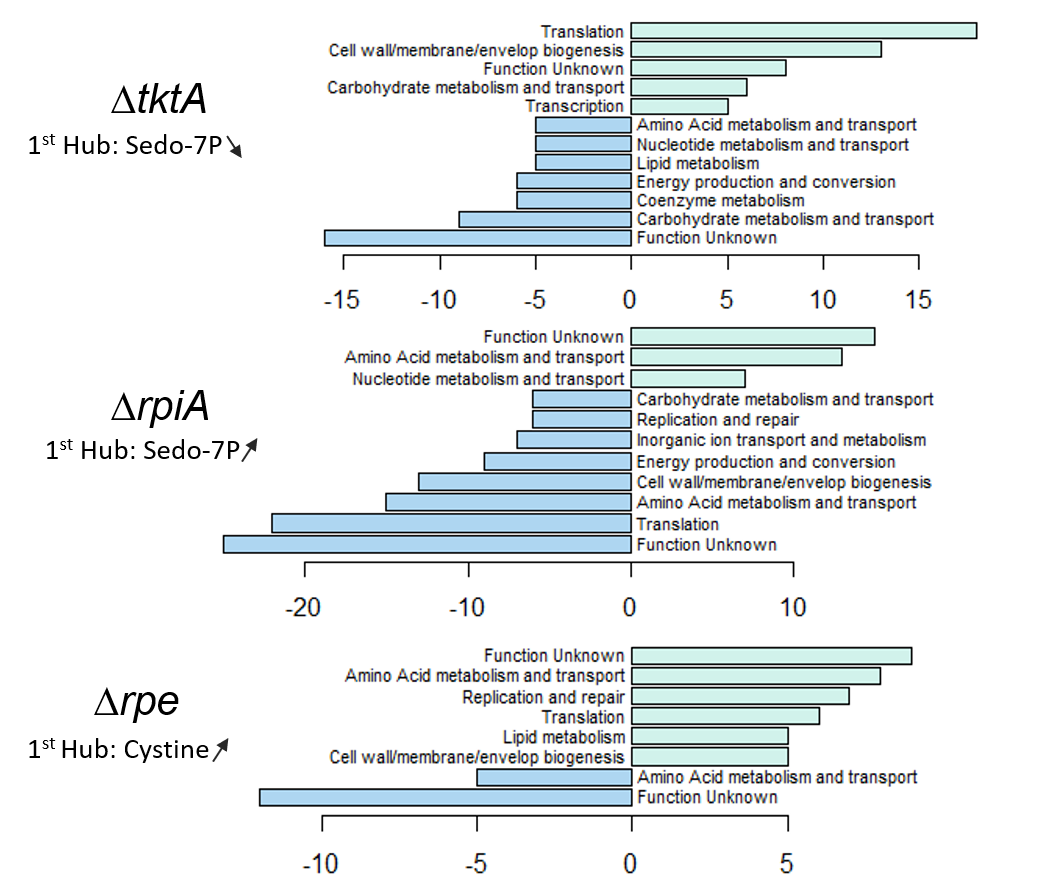

Supplement: S12 Fig — (TIF) [file ppat.1009326.s012.tif]

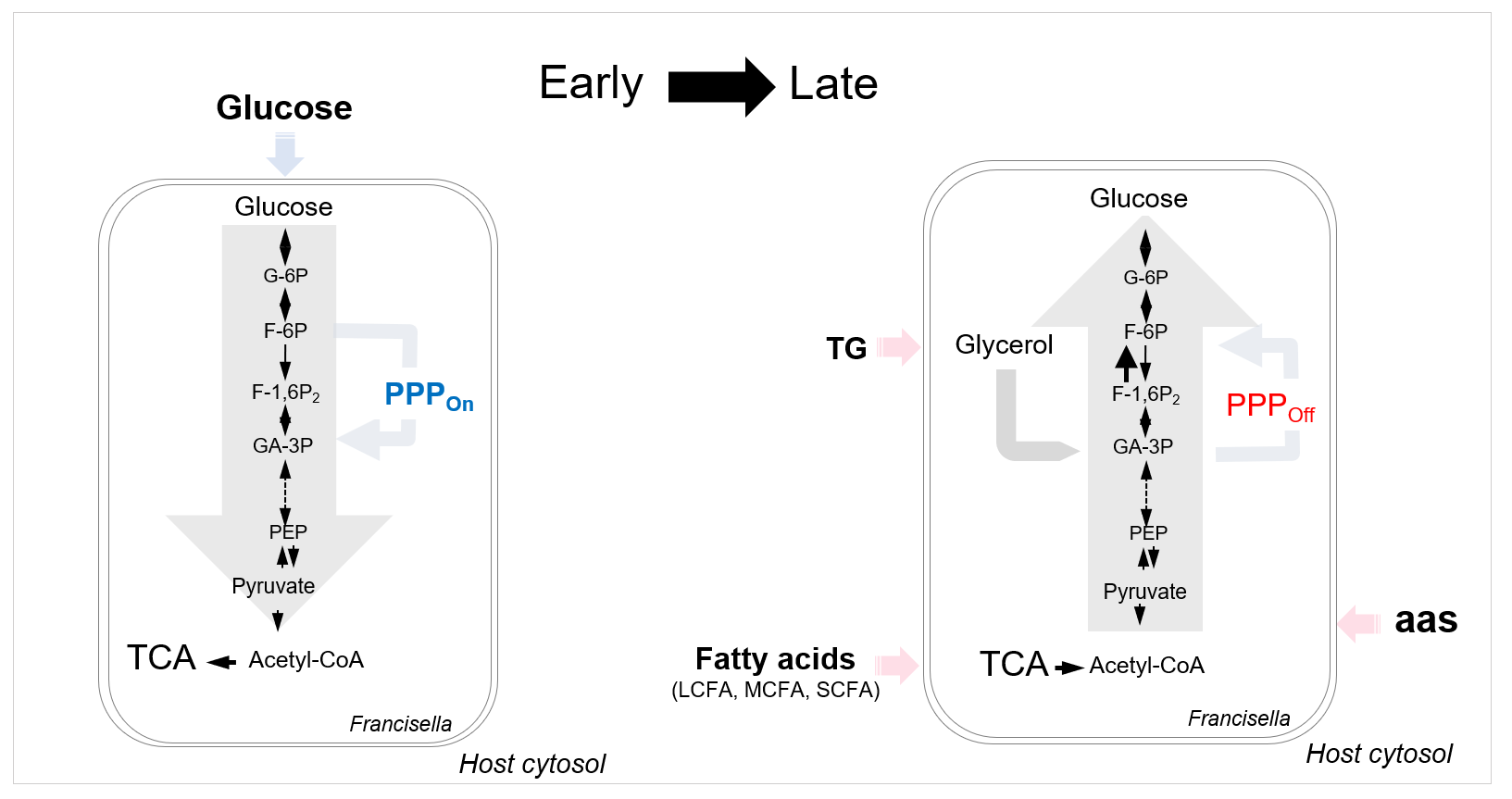

Supplement: S13 Fig — Left panel (early), during the first 24 h of the intracellular life cycle, when glucose is still available in replete condition, glycolysis (blue arrow) and the PPP are “On”. Right panel (late), at later time points (24–48 h), when glucose becomes limiting, gluconeogenesis prevails (pink arrow) and the PPP is no longer used “Off”. Different alternative carbons sources are then used to feed the gluconeogenic pathway such as: amino acids (aas); fatty acids (long chain with aliphatic tails of 13 to 21 carbons; medium chain with aliphatic tails of 6–12 carbons; and short chain fatty acids with aliphatic tails of 2–6 carbons), designated LCFA, MCFA, SCFA respectively; or triglycerides (TG). (TIF) [file ppat.1009326.s013.tif]

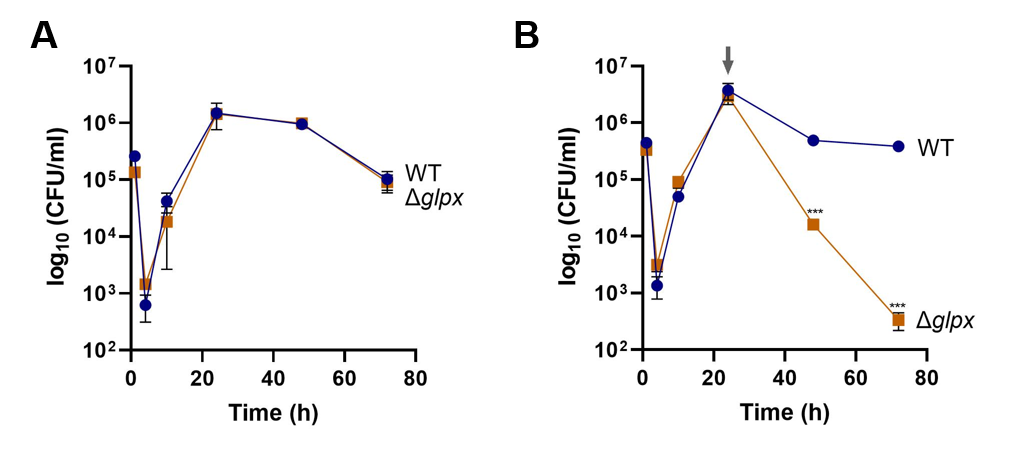

Supplement: S14 Fig — Kinetics of intracellular multiplication of the mutants was monitored in J774.1 macrophages over a 72 h-period in DMEM supplemented with (A) glucose or (B) substitution of glucose with glycerol after 24h of infection and compared to that in the wild-type F. novicida (WT). Grey arrow: change to glycerol. ***, P <0.001 (compared to WT strain; as determined by two-way ANOVA test). (TIF) [file ppat.1009326.s014.tif]

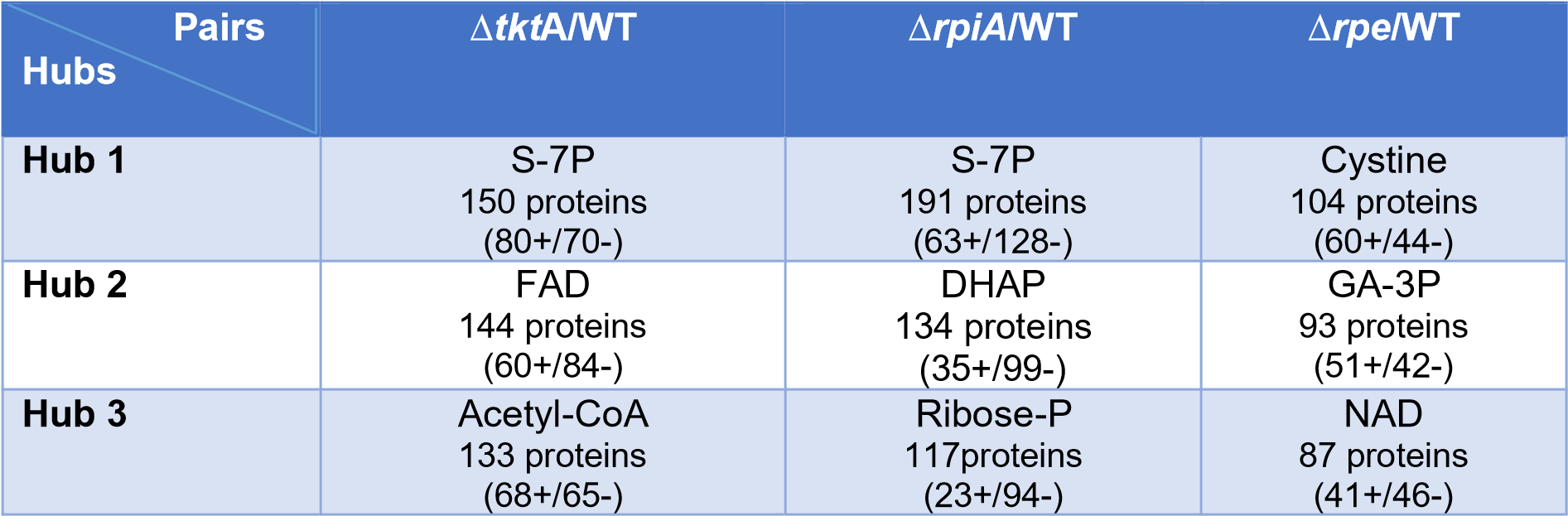

Supplement: S5 Table — (DOCX) [file ppat.1009326.s019.docx]
