# Supplementary material for: The pentose phosphate pathway constitutes a major metabolic hub in pathogenic Francisella
Source: PLoS Pathog. 2021 Aug 2;17(8):e1009326. doi: 10.1371/journal.ppat.1009326 (PMC8360588; doi:10.1371/journal.ppat.1009326)
Supplement: S2 Table — (DOCX) [file ppat.1009326.s016.docx]

| Strain name | Relevant genotype | ^a^ Ab-R | Relevant feature | ^b^ Ref |
| --- | --- | --- | --- | --- |
| ***F. novicida*** |  |  |  |  |
| U112 pKK214 | U112 (pKK214) | Tet | U112 containing empty plasmid pKK214 | 1 |
| U112 pKK-GFP | U112 (pKK214/pGro-*gfp*) | Tet | U112 transformed with pKK214 carrying *gfp* gene under pGro promoter control | 1 |
| Δ*rpiA* | U112 *rpiA*::Tn | Km | U112 with transposon insertion in *rpiA*  (tnfn1-pw060328p07q169. BEI : NR_6374) | 2 |
| Δ*rpiA*-Cp *rpiA* | Δ*rpiA* (pKK214/pGro-*rpiA*_cp_) | Km, Tet | Δ*rpiA* transformed with pKK214 carrying wild-type *rpiA* gene under pGro promoter control | 3 |
| Δ*rpiA*- pKK-GFP | Δ*rpiA* (pKK214/pGro-*gfp*_cp_) | Km, Tet | Δ*rpiA* transformed with pKK214 carrying *gfp* gene under pGro promoter control | 3 |
| Δ*rpe* | U112 *rpe*::Tn | Km | U112 with transposon insertion in *rpe*  (tnfn1-pw060419p02q104. BEI : NR_6981) | 2 |
| Δ*rpe-Cp rpe* | Δ*rpe* (pKK214/*rpe*_cp_) | Km, Tet | U112 Δ*rpe* transformed with pKK214  carrying wild-type *rpe* gene under the control of its natural promoter | 3 |
| Δ*rpe*- pKK-GFP | Δ*rpe* (pKK214/pGro-*gfp*) | Km, Tet | Δ*rpe* transformed with pKK214 carrying *gfp* gene under pGro promoter control | 3 |
| Δ*tktA* | U112 Δ*tktA*::pGro-*nptII* | Km | Replacement of *tktA* gene by *nptII* under pGro promoter control | 3 |
| Δ*tktA-Cp tktA* | Δ*tktA* (pKK214/*tktA*_cp_) | Km, Tet | Δ*tktA* transformed with pKK214 carrying wild-type *tktA* gene under the control of its natural promoter | 3 |
| Δ*tal* | U112 Δ*tal*::Tn5 | Km | *U112* with transposon insertion in *tal*  (tnfn1_pw060323p07q189. BEI :NR_5626) | 2 |
| Δ*tal*- pKK-GFP | Δ*ta*l (pKK214/pGro-*gfp*) | Km, Tet | Δ*tal* transformed with pKK214 vector carrying *gfp* gene under pGro promoter control | 3 |
| Δ*fpi* | U112 Δ*fp*i -*nptII* | Km | Replacement of *fpi* by *nptII* gene | 1 |
| Δ*fpi*- pKK-GFP | Δ*fpi* (pKK214/pGro-*gfp*) | Km, Tet | U112 Δ*fpi* transformed with pKK214 carrying *gfp* gene under pGro promoter control | 1 |
| Δ*glpx* | U112 Δ*fp*i -*nptII* | Km | Replacement of *glpx* by *nptII* gene | 1 |
| ***E. coli*** |  |  |  |  |
| *E. coli* TOP10 | F-*mcrA*, Δ(*mrr*-*hsdRMS-mcrBC*), *lacZ*ΔM15, *recA1,* Str^R^ |  | Chemically competent cell used for routine  cloning | 4 |
| *Ec* pKK | *E. coli* (pKK214) | Tet | *E. coli* TOP10 transformed with pKK214 vector | 1 |
| **Plasmids** |  |  |  |  |
| pKK | pKK214 | Tet | Empty plasmid vector pKK214 | 1 |
| pKK-GFP | pKK214/pGro-*gfp* | Tet | pKK214 carrying *gfp* gene under pGro promoter control | 1 |
| pKK*-rpiA*_cp_ | pKK214/pGro-*rpiA*_cp_ | Tet | pKK214 carrying wild-type r*piA* gene under pGro promoter control | 3 |
| pKK*-rpe*_cp_ | pKK214/*rpe*_cp_ | Tet | pKK214 carrying wild-type r*pe* gene under the control of its natural promoter | 3 |
| pKK*-tktA*_cp_ | pKK214/*tktA*_cp_ | Tet | pKK214 carrying wild-type *tktA* gene under the control of its natural promoter | 3 |
|  |  |  |  |  |

**Table S2A. Strains and plasmids**

^a^ Antibiotic resistance (Ab-R) Km: Kanamycin (10 μg.mL-1), Tet: Tetracyclin (5 μg.mL-1)

^b^ [1] lab collection, [2] kindly provided by Anders Sjöstedt (BEI ressources) [3] This study, [4] Life Technology

**Table S2B. Primers**

| Primer name | Sequence (5’-3’)^a^ | Relevant Features |
| --- | --- | --- |
| pGro FW | TTGTATGGATTAGTCGAGC | Amplification of the Kmr cassette |
| pGro(spl_nptII) RV | TTCAATCATAACAATCTTACTCCTTTGTTAAAT |  |
| nptII(spl_pGro) FW | AAGATTGTTATGATTGAACAAGATGGATTG |  |
| nptII RV | TCAGAAGAACTCGTCAAGAAGGCG |  |
| tktA upstream FW | TGTTGTACAGCCATTTTAGATAACCTCG | Construction of the Δ*tktA* mutant |
| tktA upstream(spl_K7) RV | TAATCCATACAAACTAGTATGATGATACAGTAGAGACTTTC |  |
| tktA downstream(spl_K7) FW | GAGTTCTTCTGAAAACTTTCGCAAGATAATATCTTAAGGAGA |  |
| tktA downstream RV | GTACAAGAAGCCGCTGATATGATT |  |
| tktA [SmaI] fw | TGCACCCGGGTCTGATTGAGTTGTTCGATGGTTATC | Amplification of *tktA* gene from *F. novicida* U112 used for functional complementation |
| tktA [PstI] RV | CTACTGCAGTTAGTGGTGATGGTGATGATGAACATATTTAGCAACAATATTGCTAATA |  |
| rpe [SmaI] fw | TGCACCCGGGCATTGGCATTAATCCCATGATCTCAG | Amplification of *rpe* gene from *F. novicida* U112 used for functional  complementation |
| rpe [PstI] RV | CTACTGCAGTTAGACTTTATTGAGCTCATCT |  |
| pGro[SmaI] FW | TGCACCCGGGCGACGAACTAATACTCTATCTTGTAATG | Amplification of *pGro* and *rpiA*  gene from *F.novicida* U112 used for functional  complementation |
| pGro RV | TGACAATATAGATAATCATACATGCTTGTATGGATTAGTCGAGCTAAAAAGCTCAT |  |
| rpiA fw (pGro) | ATGAGCTTTTTAGCTCGACTAATCCATACAAGCATGTATGATTATCTATATTGTCA |  |
| rpiA [PstI] RV | CTACTGCAG-TTAAAGTACAACAATATTACTATCTTT |  |
| helicase FW | CGCAGTATCTTTGGTCGTAGTAG | qRT-PCR primers |
| helicase RV | GGTTTAGCCGCTGTAAACTCTA |  |
| tktA FW | TAGGGCGTATGGTTGGCTG |  |
| tktA RV | TTGCTGCTGAGAATGGGCTT |  |
| gapA FW | AGTTAGATGGGGCTGCTCAG |  |
| gapA RV | CAGCTTTCATCGCGGCATTA |  |
| fadA fw | ACCGAAGATGAAGGCGCTAG |  |
| fadA RV | CCAGCAGTAACAGATCCACCA |  |
| fadB FW | TTGTTGCAGCTGTCAAAGGC |  |
| fadB RV | GCAGCTTCAACAAGGCCAAT |  |
| fadD FW | TGGATTCTGGAGCTTGCCTG |  |
| fadD RV | TTGTGCATCAAGGTAGCCCA |  |
| fadE FW | ACTTGGACCTGGTGAGCTTC |  |
| fadE RV | TGTTAGGGCAAAGCAAGGGA |  |

^a^ restriction sites are underlined
